# Efficient and Robust Transfer Learning of Optimal Individualized Treatment Regimes with Right-Censored Survival Data

**Published:** 2025

**Authors:** Pan Zhao, Julie Josse, Shu Yang

**Affiliations:** Statistical Laboratory, University of Cambridge; PreMeDICaL, Inria Montpellier; Department of Statistics, North Carolina State University

**Keywords:** covariate shift, data integration, policy learning, semiparametric theory, transportability

## Abstract

An individualized treatment regime (ITR) is a decision rule that assigns treatments based on patients’ characteristics. The value function of an ITR is the expected outcome in a counterfactual world had this ITR been implemented. Recently, there has been increasing interest in combining heterogeneous data sources, such as leveraging the complementary features of randomized controlled trial (RCT) data and a large observational study (OS). Usually, a covariate shift exists between the source and target population, rendering the source-optimal ITR not optimal for the target population. We present an efficient and robust transfer learning framework for estimating the optimal ITR with right-censored survival data that generalizes well to the target population. The value function accommodates a broad class of functionals of survival distributions, including survival probabilities and restrictive mean survival times (RMSTs). We propose a doubly robust estimator of the value function, and the optimal ITR is learned by maximizing the value function within a pre-specified class of ITRs. We establish the cubic rate of convergence for the estimated parameter indexing the optimal ITR, and show that the proposed optimal value estimator is consistent and asymptotically normal even with flexible machine learning methods for nuisance parameter estimation. We evaluate the empirical performance of the proposed method by simulation studies and a real data application of sodium bicarbonate therapy for patients with severe metabolic acidaemia in the intensive care unit (ICU), combining a RCT and an observational study with heterogeneity.

## 1. Introduction

Data-driven individualized decision making has recently received increasing interest in many fields, such as precision medicine (Kosorok and Laber, 2019; Tsiatis et al., 2019), mobile health (Trella et al., 2022), precision public health (Rasmussen et al., 2020) and econometrics (Belloni et al., 2017; Athey and Wager, 2021). The goal of optimal ITR estimation is to learn a decision rule that assigns the best treatment among possible options to each patient based on their individual characteristics in order to optimize some functional of the counterfactual outcome distribution in the population of interest, also known as the value function. The optimal ITR is the one with the maximal value function, and the value function of the optimal ITR is the optimal value function.

For completely observed data without censoring, one prevailing line of work in the statistical and biomedical literature uses model-based methods to solve the optimal ITR problem, such as Q-learning (Robins, 2004; Qian and Murphy, 2011; Laber et al., 2014) and A-learning (Murphy, 2003; Schulte et al., 2014; Shi et al., 2018). Alternatively, direct model-free or policy search methods have been proposed recently, including the classification perspective (Zhang et al., 2012a,b; Zhao et al., 2012; Rubin and van der Laan, 2012) and interpretable tree or list-based ITRs (Laber and Zhao, 2015; Zhang et al., 2015, 2018a), among others (Chernozhukov et al., 2019; Colangelo and Lee, 2023). In clinical studies, right-censored survival data are frequently observed as primary outcomes. Recent extensions of optimal ITR with survival data have been established in Goldberg and Kosorok (2012); Cui et al. (2017); Jiang et al. (2017); Bai et al. (2017); Díaz et al. (2018); Zhou et al. (2023). It is worth noting that learning the optimal ITR is closely linked to the estimation of conditional average treatment effects, an area that has seen significant growth recently (Hatt et al., 2022; Guo et al., 2022; Kato et al., 2024; Demirel et al., 2024a).

Researchers have investigated using machine learning algorithms to estimate the optimal ITR from large classes, which cannot be indexed by a finite-dimensional parameter (Luedtke and van der Laan, 2016a,b). One typical instance is that the optimal ITR can be learned from the blip function, which is defined as the additive effect of a blip in treatment on a counterfactual outcome, conditional on baseline covariates (Robins, 2004); and most existing regression or supervised learning methods can be directly applied (Künzel et al., 2019). However, the ITRs learned by machine learning methods can be too complex to inform policy-making and clinical practice; to facilitate the integration of data-driven ITRs into practice, it is crucial that estimated ITRs be interpretable and parsimonious (Zhang et al., 2015).

While policy learning and evaluation under distributional shift have been investigated extensively in the machine learning literature, recently there has been increasing interest in combining heterogeneous data sources, such as leveraging the complementary features of RCT data and a large OS (Rudolph and van der Laan, 2017; Yang et al., 2023a). For example, in biomedical studies and policy research, RCTs are deemed as the gold standard for treatment effects evaluation. However, due to inclusion or exclusion criteria, data availability, and study design, the enrolled participants in RCT who form the source sample may have systematically different characteristics from the target population. Therefore, findings from RCTs cannot be directly extended to the target population of interest (Cole and Stuart, 2010; Dahabreh and Hernán, 2019). On the other hand, OSs are often representative of real-world patient populations, but may be subject to confounding bias. See also Colnet et al. (2024) and Degtiar and Rose (2023) for detailed reviews. Heterogeneity in the populations is of great relevance, and a *covariate shift* usually exists where the covariate distributions differ between the source and target populations; thus, the optimal ITR for the source population is not necessarily optimal for the target population. Zhao et al. (2019) uses data from a single trial study and proposes a two-stage procedure to derive a robust and parsimonious rule for the target population; Mo et al. (2021) proposes a distributionally robust framework that maximizes the worst-case value function under a set of distributions that are “close” to the training distribution; Kallus (2021) tackles the lack of overlap for different actions in policy learning based on retargeting; Wu and Yang (2022) and Chu et al. (2023) develop a calibration weighting framework that tailors a targeted optimal ITR by leveraging the individual covariate data or summary statistics from a target population; Sahoo et al. (2022) uses distributionally robust optimization and sensitivity analysis tools to learn a decision rule that minimizes the worst-case risk incurred under a family of test distributions. However, these methods focus on continuous or binary outcomes and only consider a single sample for worst-case risk minimization; the extension to right-censored survival outcomes within the data integration context has not been studied.

We have the following contributions. We propose a new transfer learning method of finding an optimal ITR from a restricted ITR class under the super population framework where the source sample is subject to selection bias and the target sample is representative of the target population with a known sampling mechanism. Specifically, in our value search method, the value function accommodates a broad class of functionals of survival distributions, including survival probabilities and RMSTs.

We characterize the efficient influence function (EIF) (Newey, 1990, 1994) of the value function and propose the augmented estimator, which involves models for the survival outcome, propensity score, censoring and sampling processes. The proposed estimator is doubly robust in the sense that it is consistent if either the survival outcome model or the models of the propensity score, censoring, and sampling are correctly specified and is locally efficient when all models are correct. We also consider flexible data-adaptive machine learning algorithms to estimate the nuisance parameters and use the cross-fitting procedure to draw valid inferences under mild regularity conditions and a certain rate of convergence conditions (Semenova and Chernozhukov, 2020; Chernozhukov et al., 2022b).

As we consider a restricted class of ITRs indexed by a Euclidean parameter η, we also establish the cubic convergence rate of $\overset{ˆ}{\eta }$, even though its resultant limiting distribution is not standard, and thus very challenging to characterize. Based on this rate of convergence, we show that the proposed estimator for the target value function is consistent and asymptotically normal, even with flexible machine learning methods for nuisance parameter estimation. For causal ITR parameters identified by moment functions that depend on a first step unknown function, the “auto-DML” approach is a promising future direction (Farrell et al., 2021; Chernozhukov et al., 2022a).

Interestingly, when the covariate distributions of the source and target populations are the same, i.e., no covariate shift, the semiparametric efficiency bounds of our method and the standard doubly robust method (Bai et al., 2017) are equal. Moreover, if the true optimal ITR belongs to the restricted class of ITRs, the standard doubly robust method can still learn the optimal ITR despite the covariate shift, but only our method provides valid statistical inference for the value function.

## 2. Statistical Framework

### 2.1 Causal Survival Analysis

Let X denote the p-dimensional vector of covariates that belongs to a covariate space $𝒳\subset R^{p},A\in 𝒜=\{0,\;1\}$ denote the binary treatment, and $T\in R^{+}$ denote the *survival time* to the event of interest. In the presence of right censoring, the outcome T may not be observed. Let $C\in R^{+}$ denote the censoring time and Δ=I{T≤C} where I{·} is the indicator function. Let U=min{T,C} be the observed outcome, N(t)=I{U≤t,Δ=1} the counting process, and Y(t)=I{U≥t} the at-risk process.

We use the potential outcomes framework (Splawa-Neyman et al., 1990; Rubin, 1974), where for a∈𝒜={0,1}, T(a) is the survival time had the subject received treatment a. The common goal in causal survival analysis is to identify and estimate the counterfactual quantity E[y(T(a))] for some deterministic transformation function y(·). Such transformations include y(T)=min(T,L) for the RMST with some pre-specified maximal time horizon L, and y(T)=I{T≥t} for the survival probability at time t.

Under the standard assumptions (a) consistency: T=T(A), (b) positivity: Pr(A=a|X)>0 for every a∈A *almost surely*, (c) unconfoundedness: A⊥{T(1),T(0)}|X, (d) conditionally independent censoring: C⊥{T(1),T(0)}|{X,A}, we can nonparametrically identify E[y(T(a))] by the outcome regression (OR) formula or the inverse probability weighting (IPW) formula (van der Laan and Robins, 2003).

### 2.2 ITR and Value Function

Without loss of generality, we assume that larger values of T are more desirable. Typically we aim to identify and estimate an ITR d(x):𝒳→𝒜, which is a mapping from the covariate space 𝒳 to the treatment space 𝒜={0,1}, that maximizes the expected outcome in a counterfactual world had this ITR been implemented. Suppose 𝒟 is the class of candidate ITRs of interest, then define the potential outcome T(d) under any d∈𝒟 by T(d)=d(X)T(1)+(1−d(X))T(0), and the value function (Manski, 2004) of d is defined by V(d)=E[y(T(d))]. Then by maximizing V(d) over 𝒟, the optimal ITR is defined by $d^{\text{opt}}=arg\;{max}_{d\in 𝒟}\;V(d)$. See Qian and Murphy (2011) for more details.

To estimate the value function, we can use the OR or IPW formulas, and also a doubly robust method (Bai et al., 2017):

(1) $$V_{DR}(d)\;=\;E\left[\frac{I\{A\;=\;d(X)\}\Delta y(U)}{Pr(A\;=\;d(X)|X)S_{C}(U|A,X)}\right.\;\;+\left(1-\frac{I\{A\;=\;d(X)\}}{Pr(A\;=\;d(X)|X)}\right)E[y(T)|A\;=\;d(X),X]\;\;\left.+\frac{I\{A\;=\;d(X)\}}{Pr(A\;=\;d(X)|X)}\int_{0}^{\infty }\;\frac{dM_{C}(u|A,X)}{S_{C}(u|A,X)}E[y(T)|T\geq u,A,X]\right]$$

where $S_{C}(t|a,x)=Pr(C>t|A=a,X=x)$ is the conditional survival function for the censoring process, $dM_{C}(u|A=a,X)=dN_{C}(u)-Y(u)d\Lambda_{C}(u|A=a,X)$ is the martingale increment for the censoring process, $N_{C}(u)=I\{U\leq u,\Delta =0\}$ and $\Lambda_{C}(u|A=a,X)=-log\left(S_{C}(u|A=a,X)\right)$. The first term in (1) is the IPW formula, and the augmentation terms capture additional information from the subjects who do not receive treatment d, and who receive treatment d but are censored.

In (clinical) practice, it is usually desirable to consider a class of ITRs indexed by a Euclidean parameter $\eta ={\left(\eta_{1},\ldots ,\eta_{p+1}\right)}^{T}\in R^{p+1}$ for feasibility and interpretability, which has become the major trend (Rudin, 2019; Wallace and Moodie, 2015; Chen et al., 2017). Let $V(\eta )=V\left(d_{\eta }\right)$. Throughout, we focus on such a class of linear ITRs:

$${𝒟}_{\eta }=\left\{d_{\eta }:d_{\eta }(X)=I\left\{\eta^{T}\tilde{X}\geq 0\right\},\left|\eta_{p+1}\right|=1\right\},$$

where $\tilde{X}={\left(1,X^{T}\right)}^{T}$, and for identifiability we assume there exists a continuous covariate whose coefficient has absolute value one (Zhou et al., 2023); without loss of generality, we assume $\left|\eta_{p+1}\right|=1$. Therefore, the population parameter $\eta^{*}$ indexing the optimal ITR is $\eta^{*}=arg{\;max}_{\eta \in \left\{\eta \in R^{p+1}:\left|\eta_{p+1}\right|=1\right\}}\;V(\eta )$, and the optimal value function is $V\left(\eta^{*}\right)$.

#### Remark 1

Despite that we focus on linear ITRs, we note that our main identification and semiparametric efficiency results hold for general ITRs. When there is no restrictions on the ITR class 𝒟, Zhang et al. (2012b) have shown that the optimal ITR, determined by the sign of the conditional average treatment effects (CATEs), equivalently maximizes the value function. That is, the optimal ITR only depends on the CATEs, or the conditional distributions of the outcome given the covariates. But when the ITR class 𝒟 is restricted, the optimal ITR would also hinge on the covariate distribution, so we have to account for the covariate shift. The desirable interpretability and parsimony of linear ITRs can only be achieved at the expense of making suboptimal decisions for some individuals. Maronge et al. (2023) propose a reluctant additive model for interpretable nonlinear ITRs, which addresses the tension between interpretability and accuracy of treatment decisions. To prevent the estimated optimal ITR from being suboptimal or even detrimental to certain disadvantaged subpopulations, Fang et al. (2023) propose new fairness criteria that guarantee the tail performance exceed a prespecified threshold. We leave it for future research to integrate these methods into our transfer learning framework.

### 2.3 Transfer Learning

If we understand the conditional average treatment effects and, consequently, the optimal individualized treatment rule (ITR) for all values of X, concerns related to covariate shift become irrelevant. We advocate for the adoption of a transfer learning framework for two primary reasons: 1) As highlighted in the preceding section, simplicity in ITRs is often favored in practical scenarios. However, the efficacy of such an ITR might be compromised by covariate shifts, where there is a discrepancy in population distributions (Sugiyama and Kawanabe, 2012). 2) Our goal extends beyond merely identifying the optimal ITR; we are also keen on estimating and conducting inference on the value function for the target population. This step is crucial for assessing the effectiveness of the ITR before its practical application.

Instead of minimizing the worst-case risk over a single data sample, here we combine the source and target samples. This is a common practice in many clinical settings, for instance generalizing an active-controlled trial’s intention-to-treat effect where only baseline covariates are measured, from historical trial data where complete information is available (He et al., 2024). Suppose that a source sample of size n and a target sample of size m are sampled independently from the target super population with different mechanisms. Let $I_{S}$ and $I_{T}$ denote the indicator of source and target samples, respectively. Typically, the source data may originate from a randomized controlled trial (RCT), and thus it may be considered as sampled with a selection bias, meaning that its distribution differs from that in the broader target population. Frequently, the target data is uniformly sampled (or with known design weights) from this broader target population, ensuring that their distributions align. However, our approach of integrating two data sources enables us to introduce innovative weighting schemes or designs aimed at enhancing generalization.

More formally, a covariate shift means that $Pr\left(I_{S}=1|X\right)\neq Pr\left(I_{T}=1|X\right)$. In the source sample, independent and identically distributed (i.i.d.) data ${𝒪}_{s}=\left\{X_{i},A_{i},U_{i},\Delta_{i},I_{S,i}=\right.{\left.1,I_{T,i}=0\right\}}_{i=1}^{n}$ are observed from n subjects; in the target sample, it is common that only the covariates information is available, so i.i.d. data ${𝒪}_{t}={\left\{X_{i},I_{S,i}=0,I_{T,i}=1\right\}}_{i=n+1}^{n+m}$ are observed from m subjects.

In this framework, we assume that the source and target sampling mechanisms are independent, which holds if two separate studies are conducted independently by different research projects in different locations or in two separate time periods, and the target population is sufficiently large. In the context of combining the RCT and observational study, this framework corresponds to the *non-nested* study design (Dahabreh et al., 2021).

#### Remark 2

We consider the two independent samples from a (target) super population that describes the distribution of all subjects of interest to whom we intend to assign the treatment. We present the identification formulas in Section 3; however, we do not require N to be fixed and known. Equivalently, it is also possible to assume a pooled population consisting of a source population and a target population, and similar identification formulas can be proposed based on the density ratio of the two populations. Given the sampling mechanism of our data structure, we have that n and m scale at the same rate. So in our main asymptotic analysis, we can simply let n,m→∞. In the Appendix F, we analyze the asymptotic properties of our proposed methods when m or n diverges faster.

## 3. Methodology

### 3.1 Identification and Semiparametric Efficiency

To identify the causal effects from the observed data, we make the following assumptions.

#### Assumption 1

(a) T=T(A) almost surely. (b) $Pr\left(A=a|X,I_{S}=1\right)>0$ for every a almost surely. (c) $A⊥\{T(1),T(0)\}|\left\{X,I_{S}=1\right\}$. (d) $C⊥\{T(1),T(0)\}|\left\{X,A,I_{S}=1\right\}$. (e) The transformation y(⋅) admits a maximal horizon 0<h<∞, such that y(t)=y(h) for all t≥h. $Pr\left(C<h|X,A,I_{S}=1\right)<1$.

Assumption 1 includes the standard assumptions as we have introduced in Section 2.1. Here we only assume them in the source population. Assumption 1 (a) implies that the observed outcome is the potential outcome under the actual assigned treatment. Assumption 1 (b) states that each subject has a positive probability of receiving both treatments. Assumption 1 (c) requires that all confounding factors are measured so that treatment assignment is as good as random conditionally on X. Assumption 1 (d) essentially states that the censoring process is non-informative conditionally on X. Furthermore, we require additional assumptions for the source and target populations. Assumption 1 (e) says that the transformation is indifferent to survival beyond some maximal time horizon.

#### Assumption 2 (Survival mean exchangeability)

$E\left[y(T(a))|X,I_{S}=1\right]=E[y(T(a))|X]$ for every a∈𝒜.

#### Assumption 3 (Positivity of source inclusion)

$0<Pr\left(I_{S}=1|X\right)<1$ almost surely.

#### Assumption 4 (Known target design)

The target sample design weight $e(x)=\pi_{T}^{-1}(x)=1/Pr\left(I_{T}=1|X=x\right)$ is known by design.

Assumption 2 is similar to the mean exchangeability over trial participation (Dahabreh et al., 2019), and is weaker than the ignorablility assumption (Stuart et al., 2011), i.e., $I_{S}⊥\{T(1),T(0)\}|X$. Assumption 3 states that each subject has a positive probability to be included in the source sample, and implies adequate *overlap* of covariate distributions between the source and target populations. This can be a strong assumption in practice. When Assumption 3 is violated, generalization can only be made to a restricted population without extrapolation. So it would help to first identify the population where we can generalize (e.g. trial eligibility criteria). One can fit a propensity score model and trim the extreme values to ensure positivity is not violated (Yang and Ding, 2018). However, it shifts the target population and complicates the estimation and inference procedure, so we leave it for future work. Another line of research focuses on falsification tests (Hussain et al., 2022, 2023; Demirel et al., 2024b), which may serve as alternatives of Assumption 3. Assumption 4 is commonly assumed in the survey sampling literature; thus the design-weighted target sample is representative of the target population. In an observational study with simple random sampling, we have $e(x)=1/Pr\left(I_{T}=1\right)$.

Under this framework, we have the following key identity that for any g(X)

(2) $$E\left[\frac{I_{S}}{\pi_{S}(X)}g(X)\right]=E\left[I_{T}\;e(X)g(X)\right]=E[g(X)],$$

where $\pi_{S}(X)=Pr\left(I_{S}=1|X\right)$ is the sampling score.

#### Proposition 3 (Identification formulas)

Under Assumptions 1 – 4, the value function V(d) can be identified by the outcome regression formula:

(3) $$V(d)=E\left[I_{T}e(X)E\left[y(T)|A=d(X),X,I_{S}=1\right]\right],$$

and the IPW formula:

(4) $$V(d)=E\left[\frac{I_{S}}{\pi_{S}(X)}\frac{I\{A\;=\;d(X)\}}{\pi_{d}(X)}\frac{\Delta y(U)}{S_{C}(U|A,X)}\right],$$

where $\pi_{d}(X)=d(X)\pi_{A}(X)+(1-d(X))\left(1-\pi_{A}(X)\right)$ with the propensity score $\pi_{A}(X)=Pr\left(A=1|X,I_{S}=1\right)$, and $S_{C}(t|a,x)=Pr\left(C>t|A=a,X=x,I_{S}=1\right)$.

Based on the identification formulas (3) and (4), we can construct plug-in estimators for V(d), using the sampling score $\pi_{S}(X)$ or design weights e(X) to account for the sampling bias. By the identity (2), the design weights $I_{T}e(X)$ in the OR formula (3) with the target sample can also be replaced by the inverse of sampling score $I_{S}/\pi_{S}(X)$ using the source sample. However, these estimators are biased if the posited models are misspecified, and extreme weights from $\pi_{S},\pi_{A}$ and $S_{C}$ usually lead to large variability.

The simple plug-in estimators based on the formulas (3) and (4) are only consistent when the corresponding models are correctly specified, which is often unrealistic in practice. Therefore, we consider a more efficient and robust approach, motivated by the efficient influence function for V(d). We use semiparametric efficiency theory to understand the lower bound and statistical difficulty of estimating the target parameter V(d). By the EIF, we can construct the optimally efficient estimator, i.e. the one with smallest variance among all regular and asymptotically linear estimators. Such estimators have also been shown to have desirable robustness against model misspecification. We refer to Kennedy (2022) for more details.

#### Proposition 4

Under Assumptions 1 – 4, the efficient influence function of V(d) is

(5) $$\phi_{d}=\frac{I_{S}}{\pi_{S}(X)}\frac{I\{A\;=\;d(X)\}}{\pi_{d}(X)}\frac{\Delta y(U)}{S_{C}(U|A,X)}-V(d)\;\;+\left(I_{T}e(X)-\frac{I_{S}}{\pi_{S}(X)}\frac{I\{A\;=\;d(X)\}}{\pi_{d}(X)}\right)\mu (d(X),X)\;\;+\frac{I_{S}}{\pi_{S}(X)}\frac{I\{A\;=\;d(X)\}}{\pi_{d}(X)}\int_{0}^{\infty }\;\frac{dM_{C}(u|A,X)}{S_{C}(u|A,X)}Q(u,A,X)$$

where $\mu (a,x)=E\left[y(T)|A=a,X=x,I_{S}=1\right]$ and $Q(u,a,x)=E[y(T)|T\geq u,A=a,X=x,I_{S}=1]$^1^.

The semiparametric EIF guides us in constructing efficient estimators combining the source and target samples. Compared to (1), this EIF captures additional covariates information from the target population via the outcome model and thus removes the sampling bias. An efficient estimation procedure is proposed in the next section, and we show that it enjoys the double robustness property, i.e., it is consistent if either the survival outcome models μ(a,x),Q(u,a,x) or the models of propensity score $\pi_{A}(x)$, sampling score $\pi_{S}(x)$ and censoring process $S_{C}(t|a,x)$ are correct. Moreover, this EIF is Neyman orthogonal in the sense discussed in Chernozhukov et al. (2018). Therefore, a cross-fitting procedure is also proposed, allowing flexible machine learning methods for the nuisance parameters estimation, and $\sqrt{n}$ rate of convergence can be achieved.

### 3.2 An Efficient and Robust Estimation Procedure

In this section, we focus on estimating the survival function $S_{d}(t)=Pr(T(d)>t)$ as the value function under ITR d. Following the asymptotic linear characterization of survival estimands in Yang et al. (2023b), our results are readily extended to a broad class of functionals of survival distributions. For instance, the value function of the RMST under ITR d is simply $\int_{0}^{L}S_{d}(t)dt$.

Based on the EIF (5), we propose an estimator for the survival function

(6) $${\overset{ˆ}{S}}_{d}(t)=\frac{1}{n\;+\;m}\sum_{i=1}^{n+m}\;\left\{\frac{I_{S,i}}{{\overset{ˆ}{\pi }}_{S}\left(X_{i}\right)}\frac{I\left\{A_{i}\;=\;d\left(X_{i}\right)\right\}}{{\overset{ˆ}{\pi }}_{d}\left(X_{i}\right)}\frac{\Delta_{i}Y_{i}(t)}{{\overset{ˆ}{S}}_{C}\left(t|A_{i},X_{i}\right)}\right.\;\;+\left(I_{T,i}e\left(X_{i}\right)-\frac{I_{S,i}}{{\overset{ˆ}{\pi }}_{S}\left(X_{i}\right)}\frac{I\left\{A_{i}\;=\;d\left(X_{i}\right)\right\}}{{\overset{ˆ}{\pi }}_{d}\left(X_{i}\right)}\right)\overset{ˆ}{S}\left(t|A=d\left(X_{i}\right),X_{i}\right)\;\;\left.+\frac{I_{S,i}}{{\overset{ˆ}{\pi }}_{S}\left(X_{i}\right)}\frac{I\left\{A_{i}\;=\;d\left(X_{i}\right)\right\}}{{\overset{ˆ}{\pi }}_{d}\left(X_{i}\right)}\int_{0}^{\infty }\;\frac{\overset{ˆ}{S}\left(t|A_{i},X_{i}\right)d{\overset{ˆ}{M}}_{C}\left(u|A_{i},X_{i}\right)}{\overset{ˆ}{S}\left(u|A_{i},X_{i}\right){\overset{ˆ}{S}}_{C}\left(u|A_{i},X_{i}\right)}\right\},$$

where $S(t|a,x)=Pr\left(T>t|A=a,X=x,I_{S}=1\right)$ is the treatment-specific conditional survival function. We posit (semi)parametric models for the nuisance parameters. Let $\pi_{A}(X;\theta )$ be the posited propensity score model, for example, using logistic regression $logit\left\{\pi_{A}(X;\theta )\right\}=\theta^{T}\tilde{X}$, where logit(x)=log{x/(1−x)}. We use the Cox proportional hazard model $\Lambda (t|A=a,X=x)=\Lambda_{0,a}(t)exp\left(\beta_{a}^{T}x\right)$ to estimate the survival functions S(t|a,x)=exp{−Λ(t|a,x)} and the cumulative baseline hazard function $\Lambda_{0,a}(t)=\int_{0}^{t}\lambda_{0,a}(u)du$ can be estimated by the Breslow estimator (Breslow, 1972). Similarly, we posit a Cox proportional hazard model for the censoring process $\Lambda_{C}(t|A=a,X=x)=\Lambda_{C0,a}(t)exp\left(\alpha_{a}^{T}x\right)$, and the cumulative baseline hazard function $\Lambda_{C0,a}(t)$ is estimated by the Breslow estimator. The sampling score estimation is discussed in the next section.

Let $\overset{ˆ}{S}(t;\eta )={\overset{ˆ}{S}}_{d_{\eta }}(t)$ be the estimated value function for the ITR class ${𝒟}_{\eta }$, then the optimal ITR is given by $d_{\overset{ˆ}{\eta }}(x)$, where $\overset{ˆ}{\eta }=arg\;{max}_{\eta }\;\overset{ˆ}{S}(t;\eta )$.

### 3.3 Calibration Weighting

To correct the bias due to the covariate shift between populations, most existing methods directly model the sampling score (Cole and Stuart, 2010), i.e., inverse probability of sampling weighting (IPSW). However, the IPSW method requires the sampling score model to be correctly specified, and it could also be numerically unstable. Alternatively, we introduce the calibration weighting (CW) approach motivated by the identity (2), which is similar to the entropy balancing method (Hainmueller, 2012).

Let g(X) be a vector of functions of X to be calibrated, such as the moments, interactions, and non-linear transformations of X. Each subject i in the source sample is assigned a weight $q_{i}$ by solving the following optimization task:

(7) $$\underset{q1,\ldots ,q_{n}}{min}\;\sum_{i=1}^{n}\;q_{i}\log q_{i},$$

(8) $$\text{subject}\;\text{to}\;q_{i}\geq 0,\sum_{i=1}^{n}\;q_{i}=1,\sum_{i=1}^{n}\;q_{i}g\left(X_{i}\right)=\tilde{g},$$

where $\tilde{g}=\sum_{i=n+1}^{n+m}e\left(X_{i}\right)g\left(X_{i}\right)/\sum_{i=n+1}^{n+m}e\left(X_{i}\right)$ is a design-weighted estimate of E[g(X)]. The objective function (7) is the negative entropy of the calibration weights, which ensures that the empirical distribution of the weights is not too far away from the uniform, such that it minimizes the variability due to heterogeneous weights. The final balancing constraint in (8) calibrates the covariate distribution of the weighted source sample to the target population in terms of g(X). By introducing the Lagrange multiplier λ, the minimizer of the optimization task is $q_{i}=exp\left\{{\overset{ˆ}{\lambda }}^{T}g\left(X_{i}\right)\right\}/\sum_{i=1}^{n}exp\left\{{\overset{ˆ}{\lambda }}^{T}g\left(X_{i}\right)\right\}$, where $\overset{ˆ}{\lambda }$ solves the estimating equation $\sum_{i=1}^{n}exp\left\{\lambda^{T}g\left(X_{i}\right)\right\}\left\{g\left(X_{i}\right)-\tilde{g}\right\}=0$. Since we only require specifying g(X), calibration weighting avoids explicitly modeling the sampling score and evades extreme weights.

Moreover, suppose that the sampling score follows a loglinear model $\pi_{S}(X;\lambda )=exp\left\{\lambda^{T}\tilde{X}\right\}$, Lee et al. (2021, 2022) show that there is a direct correspondence between the calibration weights and the estimated sampling score. We also note that if the fraction $Pr\left(I_{S}=1\right)$ is small, the loglinear model is close to the widely used logistic regression model; our simulation studies show the robustness of calibration weights.

#### Remark 5

Other objective functions can also be used for calibration weights estimation. Chu et al. (2023) considers a generic convex distance function h(q) from the Cressie and Read family of discrepancies (Cressie and Read, 1984). Thus the optimization task is ${min}_{q1,\ldots ,q_{n}}\;\sum_{i=1}^{n}h\left(q_{i}\right)$ under the constraints (8), and the correspondence between the sampling score model $\pi_{S}$ and the objective function h has also been established.

### 3.4 Cross-Fitting

Utilizing the Neyman orthogonality of EIF (5), we consider flexible machine learning methods for estimating the nuisance parameters, where we want to remain agnostic on modeling assumptions for the complex treatment assignment, survival, and censoring processes. There is extensive recent literature on nonparametric methods for heterogeneous treatment effect estimation with survival outcomes. Cui et al. (2023) extends the generalized random forests (Athey et al., 2019) to estimate heterogeneous treatment effects in a survival and observational setting. See Xu et al. (2023) for details and practical considerations. A description of the proposed cross-fitting procedure is given below (Schick, 1986; Chernozhukov et al., 2018). Throughout, we use the subscript *CF* to denote the cross-fitted version.

**Algorithm 1:** Pseudo algorithm for the cross-fitting procedure

**Step 1** Randomly split the datasets ${𝒪}_{s}$ and ${𝒪}_{t}$ respectively into K-folds with equal size such that ${𝒪}_{s}=\cup_{k=1}^{K}{𝒪}_{s,k},{𝒪}_{t}=\cup_{k=1}^{K}{𝒪}_{t,k}$. For each k∈{1,...,K}, let ${𝒪}_{s,k}^{c}={𝒪}_{s}\backslash {𝒪}_{s,k},{𝒪}_{t,k}^{c}={𝒪}_{s}\backslash {𝒪}_{t,k}$
**Step 2** For each k∈{1,...,K}, estimate the nuisance parameters only using data ${𝒪}_{s,k}^{c}$ and ${𝒪}_{t,k}^{c}$; then obtain an estimate of the value function ${\overset{ˆ}{V}}_{CF,k}(\eta )$ using data ${𝒪}_{s,k}$
**Step 3** Aggregate the estimates from K folds: ${\overset{ˆ}{V}}_{CF}(\eta )=\frac{1}{K}\sum_{k=1}^{K}\;{\overset{ˆ}{V}}_{CF,k}(\eta )$
**Step 4** The estimated optimal ITR is indexed by $\overset{ˆ}{\eta }=arg\;{max}_{\eta }\;{\overset{ˆ}{V}}_{CF}(\eta )$

## 4. Asymptotic Properties

In this section, we present the asymptotic properties of the proposed methods. To establish the asymptotic properties, we require the following assumptions.

### Assumption 5

(i) The value function V(η) is twice continuously differentiable in a neighborhood of $\eta^{*}$. (ii) There exists some constant $\delta_{0}>0$ such that $Pr(0<\left|{\tilde{X}}^{T}\eta \right|<\delta )=O(\delta )$, where the big-O term is uniform in $0<\delta <\delta_{0}$.

Condition (i) is a standard regularity condition to establish uniform convergence. Similar margin conditions as (ii), which state that $Pr(0<|\gamma (X)|<\delta )=O\left(\delta^{\alpha }\right)$^2^, are often assumed in the literature of classification (Tsybakov, 2004; Audibert and Tsybakov, 2007), reinforcement learning (Farahmand, 2011; Hu et al., 2025) and optimal treatment regimes (Luedtke and van der Laan, 2016a; Luedtke and Chambaz, 2020), to guarantee a fast convergence rate. Note that α=0 imposes no restriction, which allows γ(X)=0 almost surely, i.e., the challenging setting of exceptional laws where the optimal ITR is not uniquely defined (Robins, 2004; Robins and Rotnitzky, 2014), while the case α=1 is of particular interest and would hold if γ(X) is absolutely continuous with bounded density.

### Theorem 6

Under Assumptions 1 – 5 and standard regularity conditions provided in the Supplementary Material, if either the survival outcome model, or the models of the propensity score, the sampling score and the censoring process are correct, we have that as n,m→∞, (i) $\overset{ˆ}{S}(t;\eta )\to S(t;\eta )$ for any η and 0<t≤L; (ii) $\sqrt{n+m}\{\overset{ˆ}{S}(t;\eta )-S(t;\eta )\}$ converges weakly to a mean zero Gaussian process for any η; (iii) $\left(n+m{)}^{1/3}{\left\|\overset{ˆ}{\eta }-\eta^{*}\right\|}_{2}=O_{p}(1\right)$; (iv) $\sqrt{n+m}\left\{\overset{ˆ}{S}(t;\overset{ˆ}{\eta })-S\left(t;\eta^{*}\right)\right\}\to 𝒩(0,\sigma_{t,1}^{2})$, where $\sigma_{t,1}$ is given in the Supplementary Material.

Next, to characterize the asymptotic behavior of the estimator with the nonparametric estimation of nuisance parameters, we assume the following consistency and convergence rate conditions of the nonparametric plug-in nuisance estimators.

### Assumption 6

Assume the following consistency conditions ${\left\|{\overset{ˆ}{\pi }}_{A}(x)-\pi_{A}(x)\right\|}_{2}=o_{p}(1)$, ${\left\|{\overset{ˆ}{\pi }}_{S}(x)-\pi_{S}(x)\right\|}_{2}=o_{p}(1)$, and for a=0,1,

$$\underset{u\leq h}{sup}\;{\left\|{\overset{ˆ}{S}}_{C}(u|a,X)-S_{C}(u|a,X)\right\|}_{2}=o_{p}\left(1\right),\underset{u\leq h}{sup}\;{\left\|\frac{{\overset{ˆ}{\lambda }}_{C}\left(u|a,\;X\right)}{{\overset{ˆ}{S}}_{C}\left(u|a,\;X\right)}-\frac{\lambda_{C}\left(u|a,\;X\right)}{S_{C}\left(u|a,\;X\right)}\right\|}_{2}=o_{p}\left(1\right),\;\;\|\overset{ˆ}{\mu }\left(a,X\right)-\mu \left(a,X\right){\|}_{2}=o_{p}\left(1\right),\underset{u\leq h}{sup}\;\left\|\overset{ˆ}{Q}\left(u,a,X\right)-Q\left(u,a,X\right)\right\|=o_{p}\left(1\right),$$

and the following rate of convergence conditions: ${\left\|{\overset{ˆ}{\pi }}_{A}(x)-\pi_{A}(x)\right\|}_{2}=o_{p}\left(n^{-1/4}\right),\|{\overset{ˆ}{\pi }}_{S}(x)-\pi_{S}(x){\|}_{2}=o_{p}\left(n^{-1/4}\right)$, and for a=0,1,

$$\underset{u\leq h}{sup}\;{\left\|{\overset{ˆ}{S}}_{C}(u|a,X)-S_{C}(u|a,X)\right\|}_{2}=o_{p}\left(n^{-1/4}\right),\underset{u\leq h}{sup}\;{\left\|\frac{{\overset{ˆ}{\lambda }}_{C}(u|a,\;X)}{{\overset{ˆ}{S}}_{C}(u|a,\;X)}-\frac{\lambda_{C}(u|a,\;X)}{S_{C}(u|a,\;X)}\right\|}_{2}=o_{p}\left(n^{-1/4}\right),\;\;\|\overset{ˆ}{\mu }(a,X)-\mu (a,X){\|}_{2}=o\left(n^{-1/4}\right),\underset{u\leq h}{sup}\;\|\overset{ˆ}{Q}(u,a,X)-Q(u,a,X){\|}_{2}=o\left(n^{-1/4}\right).$$

The rate conditions in Assumption 6 are generally assumed in the literature (Kennedy, 2022). This rate can be achieved by many existing methods under certain structural assumptions on the nuisance parameters. Note that the nuisance parameters do not necessarily need to be estimated at the same rates $n^{-1/4}$ for our theorems to hold; it would suffice that the product of rates of any combination of two nuisance parameters is $n^{-1/2}$.

### Theorem 7

Under Assumptions 1 – 6, we have that as n,m→∞, (i) ${\overset{ˆ}{S}}_{CF}(t;\eta )\to S(t;\eta )$ for any η and 0<t≤L; (ii) $\sqrt{n+m}\left\{{\overset{ˆ}{S}}_{CF}(t;\eta )-S(t;\eta )\right\}$ converges weakly to a mean zero Gaussian process for any η; (iii) $\left(n+m{)}^{1/3}{\left\|\overset{ˆ}{\eta }-\eta^{*}\right\|}_{2}=O_{p}(1\right)$; (iv) $\sqrt{n+m}\left\{{\overset{ˆ}{S}}_{CF}(t;\overset{ˆ}{\eta })-S\left(t;\eta^{*}\right)\right\}\to 𝒩(0,\sigma_{t,2}^{2})$, where $\sigma_{t,2}$ is given in the Supplementary Material.

Besides the survival functions, another common measure of particular interest in survival analysis is the RMST. Let $V_{RMST}(\eta )=E\left[min\left(T\left(d_{\eta }\right),L\right)\right]$. We present two corollaries.

### Corollary 8

Under Assumptions 1 – 5 and standard regularity conditions provided in the Supplementary material, if either the survival outcome model or the models of the propensity score, the censoring and sampling processes are correct, we have that as n,m→∞, (i) ${\overset{ˆ}{V}}_{RMST}(\eta )\to V_{RMST}(\eta )$ for any η; (ii) $\left(n+m{)}^{1/3}{\left\|\overset{ˆ}{\eta }-\eta^{*}\right\|}_{2}=O_{p}(1\right)$; (iii) $\sqrt{n+m}\left\{{\overset{ˆ}{V}}_{RMST}(\overset{ˆ}{\eta })-V_{RMST}\left(\eta^{*}\right)\right\}\to 𝒩\left(0,\sigma_{3}^{2}\right)$, where $\sigma_{3}$ is given in the Supplementary Material.

### Corollary 9

Under Assumptions 1 – 6, we have that as n,m→∞, (i) ${\overset{ˆ}{V}}_{RMST,CF}(\eta )\to V_{RMST}(\eta )$ for any η; (ii) $(n+m{)}^{1/3}{\left\|\overset{ˆ}{\eta }-\eta^{*}\right\|}_{2}=O_{p}(1);\;(iii)\;\sqrt{n+m}\left\{{\overset{ˆ}{V}}_{RMST,CF}(\overset{ˆ}{\eta })-V_{RMST}\left(\eta^{*}\right)\right\}\to 𝒩\left(0,\sigma_{4}^{2}\right)$, where $\sigma_{4}$ is given in the Supplementary Material.

Finally, we show that when the covariate distributions of the source and target populations are the same, the semiparametric efficiency bounds of ${\overset{ˆ}{V}}_{DR}(\eta )$ and ${\overset{ˆ}{V}}_{CF}(\eta )$ are equal.

### Theorem 10

Under Assumptions 1 – 6, when the covariate distributions of the source and target populations are the same, both $\sqrt{n}\left\{{\overset{ˆ}{V}}_{DR}(\eta )-V(\eta )\right\}$ and $\sqrt{n+m}\left\{{\overset{ˆ}{V}}_{CF}(\eta )-V(\eta )\right\}$ are asymptotically normal with mean zero and same variance.

Theorem 10 implies that when there is no covariate shift, our proposed estimator does not lose efficiency in comparison to the original double robust estimator since the augmentation term in EIF (5) from the target population, $I_{T}e(X)\mu (d(X),X)$, is asymptotically equal to this term evaluated on the source population in this case.

Moreover, when the covariate shift exists, we consider the optimal ITR $d^{\text{opt}}$ without restriction on the ITR class.

### Theorem 11

Under Assumptions 1 – 6, If $d^{\mathrm{opt}}\in {𝒟}_{\eta }$, i.e., $d^{\mathrm{opt}}=d_{\eta^{*}},\;both\;the\;maximizers\;of\;{\overset{ˆ}{V}}_{DR}(\eta )$ and ${\overset{ˆ}{V}}_{CF}(\eta )$ converge to $\eta^{*}.\;However,\;{\overset{ˆ}{V}}_{DR}(\eta )$ is a biased estimator of V(η).

Theorem 11 implies if the true optimal ITR belongs to the restricted ITR class ${𝒟}_{\eta }$, standard methods, without accounting for the covariate shift, are still able to recover the optimal ITR but fail to be consistent for the value function, due to the covariate shift. And we can only rely on the proposed method to draw valid inferences.

## 5. Simulation

In this section, we investigate the finite-sample properties of our method through extensive numerical simulations. The R code to replicate all results is available at https://github.com/panzhaooo/transfer-learning-survival-ITR. Consider a target population of sample size $N=2\times 10^{5}$. The covariates ${\left(X_{1},X_{2},X_{3}\right)}^{T}$ are generated from a multivariate normal distribution with mean 0, unit variance with $corr\left(X_{1},X_{3}\right)=0.2$ and all other pairwise correlations equal to 0, and further truncated below −4 and above 4 to satisfy regularity conditions. The target sample is a random sample of size m=8000 from the target population. The sampling score follows $\pi_{S}(X)=expit\left(-4.5-0.5X_{1}-0.5X_{2}-0.4X_{3}\right)$; thus the source sampling rate is around 1.6%, and the source sample size around n=3000. The treatment assignment mechanism in the source sample follows $\pi_{A}(X)=expit\left(0.5+0.8X_{1}-0.5X_{2}\right)$.

The counterfactual survival times T(a) are generated according to the hazard functions $\lambda (t|A=0,X)=exp(t)\cdot exp\left(-2.5-1.5X_{1}-X_{2}-0.7X_{3}\right)$ and $\lambda (t|A=1,X)=\exp\left(t\right)\cdot exp\left(-1-X_{1}-0.9X_{2}-X_{3}-2X_{2}^{2}+X_{1}X_{3}\right)$. The censoring time C is generated according to the hazard functions $\lambda_{C}(t|A=0,X)=0.04exp(t)\cdot exp\left(-1.6+0.8X_{1}-1.1X_{2}-0.7X_{3}\right)$ and $\lambda_{C}(t|A=1,X)=0.04exp(t)\cdot exp\left(-1.8-0.8X_{1}-1.7X_{2}-1.4X_{3}\right)$. The resultant censoring rate is approximately 20%.

We consider the RMST with the maximal time horizon L=4 as the value function. To evaluate the performance of different estimators for optimal ITRs, we compute the corresponding true value functions and percentages of correct decisions (PCD) for the target population. Specifically, we generate a large sample with size $\tilde{N}=1\times 10^{5}$ from the target population. The true value function of any ITR d(·;η) is computed by $V(\eta )={\tilde{N}}^{-1}\sum_{i=1}^{\tilde{N}}min\left\{d\left(X_{i};\eta \right)T_{i}(1)+\left(1-d\left(X_{i};\eta \right)\right)T_{i}(0),L\right\}$ and its associated PCD is computed by $1-{\tilde{N}}^{-1}\sum_{i=1}^{N}\left|d\left(X_{i};\eta^{*}\right)-d\left(X_{i};\eta \right)\right|$, where $\eta^{*}=arg\;{max}_{\eta }\;V(\eta )$.

We compare the following estimators for the RMST $\overset{ˆ}{V}(\eta )=\int_{0}^{L}\overset{ˆ}{S}(t;\eta )dt$:

- Naive: ${\overset{ˆ}{S}}^{\text{Naive}}(t;\eta )=\frac{1}{n}\sum_{i=1}^{n}\frac{I\left\{A_{i}=d\left(X_{i}\right)\right\}}{{\overset{ˆ}{\pi }}_{d}\left(X_{i}\right)}\frac{\Delta_{i}Y_{i}(t)}{{\overset{ˆ}{S}}_{C}(U|A,X)}$; IPW formula (4) without using the sampling score;
- IPSW: ${\overset{ˆ}{S}}^{IPSW}(t;\eta )=\frac{1}{n}\sum_{i=1}^{n}\frac{I_{S,i}}{{\overset{ˆ}{\pi }}_{S}\left(X_{i}\right)}\frac{I\left\{A_{i}=d\left(X_{i}\right)\right\}}{{\overset{ˆ}{\pi }}_{d}\left(X_{i}\right)}\frac{\Delta_{i}Y_{i}(t)}{{\overset{ˆ}{S}}_{C}(U|A,X)}$; IPW formula (4) where the sampling score is estimated via logistic regression;
- CW-IPW: ${\overset{ˆ}{S}}^{CW-IPW}(t;\eta )=\sum_{i=1}^{n}q_{i}\frac{I\left\{A_{i}=d\left(X_{i}\right)\right\}}{{\overset{ˆ}{\pi }}_{d}\left(X_{i}\right)}\frac{\Delta_{i}Y_{i}(t)}{{\overset{ˆ}{S}}_{C}(U|A,X)}$ IPW formula (4) where the sampling score is estimated by calibration weighting;
- CW-OR: ${\overset{ˆ}{S}}^{CW-OR}(t;\eta )=\sum_{i=1}^{n}q_{i}\overset{ˆ}{S}\left(t|A=d\left(X_{i}\right),X_{i}\right)$; OR formula (3) in combination with calibration weights by the identity (2);
- ORt: ${\overset{ˆ}{S}}^{\text{ORt}}(t;\eta )=\frac{1}{m}\sum_{i=n+1}^{n+m}\overset{ˆ}{S}\left(t|A=d\left(X_{i}\right),X_{i}\right)$; OR formula (3) evaluated on the target sample;
- ACW: augmented estimator (6), where the sampling score is estimated by calibration weighting.

### Remark 12

Since the estimated value functions are non-convex and non-smooth, multiple local optimal may exist in the optimization task, and many derivatives-based algorithms do not work for this challenging setting. Here we utilize the genetic algorithm implemented in the R package rgenoud (Mebane Jr. and Sekhon, 2011), which performs well in our numerical experiments. We refer to Mitchell (1998) for algorithmic details.

### 5.1 (Semi)parametric Models

We first consider the setting where the nuisance parameters are estimated by posited (semi)parametric working models as introduced in Section 3.2. To assess the performance of these estimators under model misspecification, we consider four scenarios: (1) all models are correct, (2) only the survival outcome model is correct, (3) only the survival outcome model is wrong, (4) all models are wrong. For the wrong sampling model, the weights are estimated using calibration on $e^{X_{1}}$. The wrong propensity score model is fitted on $e^{X_{3}}$. The wrong Cox models for survival and censoring times are fitted on ${\left(e^{X_{1}},e^{X_{2}},e^{X_{3}}\right)}^{T}$.

**Figure 1:**
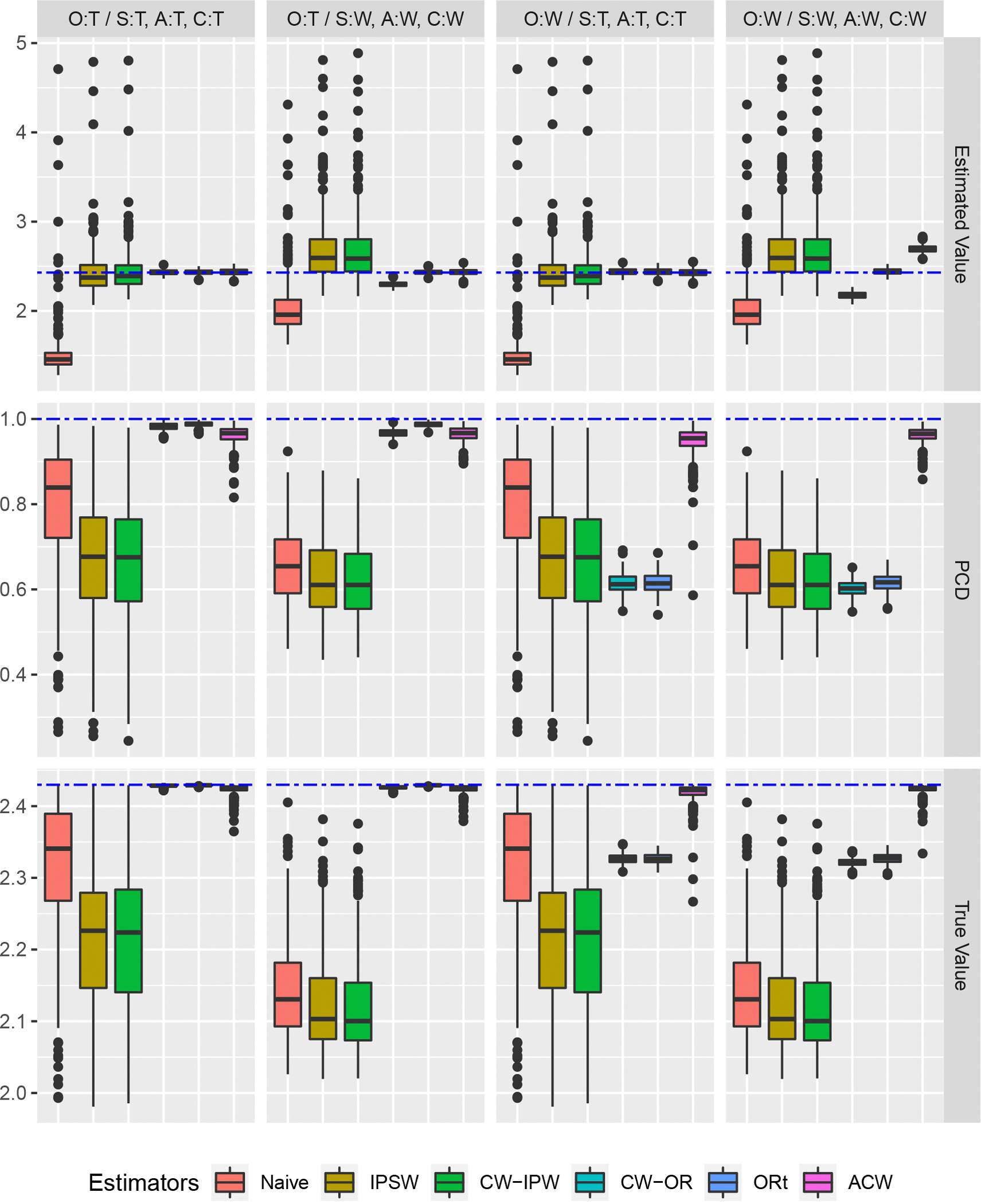
Boxplot of the estimated value, true value and PCD results of estimators under four model specification scenarios. O: survival outcome, S: sampling score, A: propensity score, C: censoring; T: True (correctly specified) model, W: Wrong (misspecified) model.

Figure 1 and Table 1 report the simulation results from 350 Monte Carlo replications. Variance is estimated by a bootstrap procedure with B=200 bootstrap replicates. The proposed ACW estimator is unbiased in scenarios (1) – (3), and the 95% coverage probabilities approximately achieve the nominal level, which shows the double robustness property. In comparison, the bias of the regression based methods is 10 times larger than our proposed method when the outcome model is misspecified, and the bias of the weighting based methods is 100 times larger than our proposed method when the models for sampling, treatment assignment and censoring are wrong.

### 5.2 Flexible Machine Learning Methods

When utilizing flexible ML methods, we construct the cross-fitted ACW estimator as introduced in Section 3.4. The data generation process is the same as above, except that the censoring time *C* is generated according to the hazard functions $\lambda_{C}(t|A=0,X)=0.2\;exp(t)\cdot exp\left(-1.6+0.8X_{1}-1.1X_{2}-0.7X_{3}\right)$ and $\lambda_{C}(t|A=1,X)=0.2\;exp(t)\cdot exp(-1.8-\left.0.8X_{1}-1.7X_{2}-1.4X_{3}\right)$ which leads to an increased censoring rate of approximately 33%, so there are enough observations to get an accurate estimate of the censoring process. The propensity score is estimated by the generalized random forest. The conditional survival and censoring functions are estimated by the random survival forest. The calibration weighting uses calibration on the first- and second-order moments of X.

First, we study the impact of sample sizes on the performance of the ML methods, and simulation results are given in the Supplementary Material. With a small sample size, the ACW estimator is largely biased, and the bias diminishes as the sample size increases.

**Figure 2:**
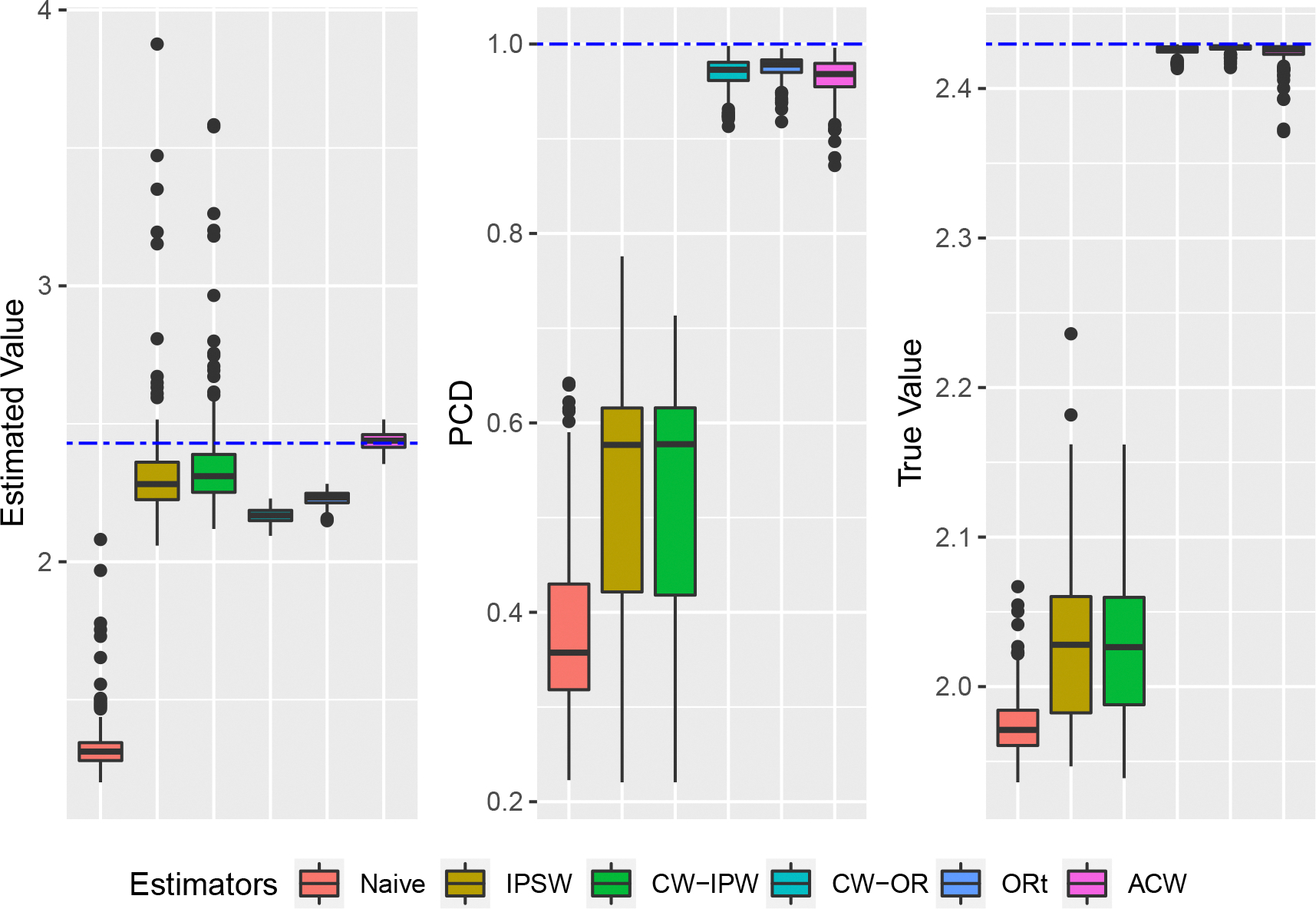
Boxplots of the estimated value, true value, and PCD of different estimators using flexible ML methods.

Next, we compare the performance of different estimators with target population size $N=6\times 10^{5}$ and target sample size m=24000. Figure 2 shows the simulation results from 200 Monte Carlo replications. The two IPW-based estimators are biased and perform poorly due to the large variability of weights. The two OR-based estimators have comparable performance as the ACW estimator in terms of PCD and true value function but still suffer from the overfitting bias. Only the ACW estimator is consistent and provides valid inferences.

## 6. Real Data Analysis

In this section, to illustrate the proposed method, we study the sodium bicarbonate therapy for patients with severe metabolic acidaemia in the intensive care unit by leveraging the RCT data BICAR-ICU (Jaber et al., 2018) and the observational study (OS) data from Jung et al. (2011). Specifically, we consider the BICAR-ICU data as the source sample and the observational study data as the target sample. The BICAR-ICU is a multi-center, open-label, randomized controlled, phase 3 trial between May 5, 2015, and May 7, 2017, which includes 387 adult patients admitted within 48 hours to the ICU with severe acidaemia. The prospective, multiple-center observational study was conducted over thirteen months in five ICUs, consisting of 193 consecutive patients who presented with severe acidemia within the first 24 hours of their ICU admission. Some heterogeneity exists between the two populations.

Both the RCT and OS datasets contain detailed measurements of ICU patients with severe acidaemia. Motivated by the clinical practice and existing work in the medical literature, we consider ITRs that depend on the following five variables: SEPSIS, AKIN, SOFA, SEX, and AGE. A detailed description of the data preprocessing and variable selection is given in the Supplementary Material. Table 2 summarizes the baseline characteristics of the two datasets. The baseline covariates distribution of the patients in the BICAR-ICU differs from the distribution in the observational study; specifically, the BICAR-ICU patients have higher SOFA scores and the more frequent presence of acute kidney injury and sepsis.

We apply our proposed ACW estimator to learn the optimal ITR for the target population. The calibration weights are estimated based on the means of continuous covariates and the proportions of the binary covariates. The propensity score is estimated using a logistic regression model, and the Cox proportional hazard model is fitted for the survival outcome with all covariates. The censoring only occurred on the 28th day when the follow-up in ICU ends. We consider the class of linear ITRs that depend on all five variables $𝒟=\left\{I\left\{\eta_{1}+\eta_{2}SEPSIS+\eta_{3}AKIN+\eta_{4}SOFA+\eta_{5}SEX+\eta_{6}AGE>0\right\}:\eta_{1},\ldots ,\eta_{6}\in R,\left|\eta_{6}\right|=1\right\}$, with the aim to maximize the RMST within 28 days in ICU stay. The estimated parameter indexing the optimal ITR is ${\overset{ˆ}{\eta }}_{ACW}=(22.9,\;-36.1,\;87.4,-9.8,\;33.7,\;1.0{)}^{T}$, which leads to an estimated value function $\overset{ˆ}{V}\left({\overset{ˆ}{\eta }}_{ACW}\right)=19.52$ days, with confidence interval [17.74,21.30] given by 200 bootstraps. In contrast, we also use the standard double robust method to estimate the optimal ITR for the RCT, indexed by ${\overset{ˆ}{\eta }}_{\text{DR.RCT}}$ which maximize the value function ${\overset{ˆ}{V}}_{\text{DR}}(\eta )$ in (1) with y(T)=min(T,28). The estimated value function is $\overset{ˆ}{V}\left({\overset{ˆ}{\eta }}_{\text{DR.RCT}}\right)=15.37$ days for the target population.

## 7. Discussion

In this paper, we present an efficient and robust transfer learning framework for estimating optimal ITR with right-censored survival data that generalizes well to the target population. The proposed method can be improved or extended in several directions for future work. Construction and estimation of optimal ITRs for multiple decision points with censored survival data are challenging, taking into account the timing of censoring, events and decision points (Jiang et al., 2017; Hager et al., 2018), e.g., using a reinforcement learning method (Cho et al., 2023). Furthermore, besides the class of ITRs indexed by a Euclidean parameter, it may be possible to consider other classes of ITRs, such as tree or list-based ITRs. The current work focus on value functions in the form V(d)=E[y(T(d))] and can also be modified in case of optimizing certain easy-to-interpret quantile criteria, which does not require specifying an outcome regression model and is robust for heavy-tailed distributions (Zhou et al., 2023). And relaxing the restrictive assumptions such as positivity (Yang and Ding, 2018; Jin et al., 2022) and unconfoundedness (Cui and Tchetgen Tchetgen, 2021; Qi et al., 2023) for learning optimal ITRs is also a fruitful direction.

## Supplementary Material

1

## Figures and Tables

**Table 1:** Numerical results under four diffierent model specification scenarios.

	Bias	SD	SE	CP(%)	Bias	SD	SE	CP(%)

	O:T / S:T, A:T, C:T				O:T / S:W, A:W, C:W			
Naive	−0.8801	0.4595	0.2189	7.43	−0.3528	0.5024	0.4598	37.43
IPSW	0.0185	0.3685	0.2562	87.14	0.3377	0.7144	0.6958	98.29
CW-IPW	0.0378	0.3701	0.2498	88.29	0.3406	0.7144	0.6957	97.71
CW-OR	0.0047	0.0273	0.0286	96.29	−0.1312	0.0269	0.0279	0.57
ORt	0.0041	0.0258	0.0262	95.14	0.0035	0.0258	0.0262	95.71
ACW	0.0070	0.0380	0.0369	94.29	0.0055	0.0316	0.0334	95.43

	O:W / S:T, A:T, C:T				O:W / S:W, A:W, C:W			
Naive	−0.8801	0.4595	0.2207	6.86	−0.3528	0.5024	0.5018	38.57
IPSW	0.0185	0.3685	0.2486	87.71	0.3377	0.7144	0.7586	99.14
CW-IPW	0.0378	0.3701	0.2418	88.86	0.3406	0.7144	0.7570	98.57
CW-OR	0.0103	0.0370	0.0362	92.29	−0.2551	0.0366	0.0391	0.00
ORt	0.0094	0.0365	0.0355	94.00	0.0115	0.0328	0.0355	95.71
ACW	0.0010	0.0426	0.0419	93.14	0.2644	0.0422	0.0475	0.57

Bias is the empirical bias of point estimates; SD is the empirical standard deviation of point estimates; SE is the average of bootstrap standard error estimates; CP is the empirical coverage probability of the 95% confidence intervals.

**Table 2:** Summary of baseline characteristics of the BICAR-ICU trial sample and the OS sample.

	SEPSIS	AKIN	SOFA	SEX	AGE

BICAR-ICU (*n* = 387)	236 (60.98%)	181 (46.77%)	10.12 (3.72)	237 (61.24%)	63.95 (14.41)
OS (*m* = 193)	99 (51.30%)	75 (38.86%)	9.10 (4.54)	122 (63.21%)	62.73 (17.49)

Mean (standard deviation) for continuous and number (proportion) for the binary covariate.

## Footnotes

1. Note that $E[y(T)|T\geq u,\;A,\;X]=-\int_{u}^{\infty }y(s)dS(s|A,\;X)/S(u|A,\;X)$. For instance, when y(T)=I{T≥t}, we have E[y(T)∣T≥u,A,X]=S(t∣A,X)/S(u∣A,X) for u≤t.

2. Let γ(X)=E[T∣A=1,X]−E[T∣A=0,X] denote the conditional average treatment effect, then the optimal ITR in an unrestricted class is given by d(X)=I{γ(X)>0}.
