# Supplementary material for: Efficient and Robust Transfer Learning of Optimal Individualized Treatment Regimes with Right-Censored Survival Data
Source: J Mach Learn Res. Author manuscript; Available in PMC 2026 Mar 11. (PMC12974684)
Supplement: 1 [file NIHMS2081881-supplement-1.pdf]

Appendix A. Preliminaries

A.1 Counting Processes for Cox Model

We use the counting process theory of Andersen and Gill (1982) in our theoretical framework to study the large sample properties of Cox model. We state the existing results that are used in our proof.

Let $X^{\otimes l}$ denote 1 for $l=0$, X for $l=1$, and XX^T for $l=2$. Define

$$U_a^{(l)}(\beta_a,t)=\frac{1}{n_a}\sum_{i=1}^{n} I\{A_i=a\}X_i^{\otimes l}\exp(\beta_a^T X_i)Y_i(t) \text{ and } u_a^{(l)}(\beta_a,t)=\mathbb{E}\left[X^{\otimes l}\exp(\beta_a^T X)Y(t)\right],$$

where $n_a=\sum_{i=1}^n I\{A_i=a\}$, and define

$$E_a(\beta_a,t)=\frac{U_a^{(1)}(\beta_a,t)}{U_a^{(0)}(\beta_a,t)} \text{ and } e_a(\beta_a,t)=\frac{u_a^{(1)}(\beta_a,t)}{u_a^{(0)}(\beta_a,t)}.$$

The maximum partial likelihood estimator $\hat\beta_a$ for the Cox proportional hazards model solves the estimating equation

$$\mathcal{S}_{a,n}(\beta_a)=\frac{1}{n_a}\sum_{i=1}^{n} I\{A_i=a\}\int\left\{X_i-\frac{U_1^{(1)}(\beta_a,u)}{U_1^{(0)}(\beta_a,u)}\right\}\mathrm{d}N_i(u)=0,$$

and the cumulative baseline hazard function $\hat\Lambda_{0,a}$ is estimted by the Breslow estimator:

$$\hat\Lambda_{0,a}(t)=\int_0^t \frac{\sum_{i=1}^n I\{A_i=a\}\mathrm{d}N_i(u)}{\sum_{i=1}^n I\{A_i=a\}\exp(\hat\beta_a^T X_i)Y_i(u)},\, a=0,1.$$

Under certain regularity conditions (Andersen and Gill, 1982, Conditions A – D), $\hat\beta_a$ and $\hat\Lambda_{0,a}$ converge in probability to the limits β_a^* and $\Lambda_{0,a}^*$, respectively; and we have

$$\sqrt{n_a}(\hat\beta_a-\beta_a^*)=\Gamma_a^{-1}\frac{1}{\sqrt{n_a}}\sum_{i=1}^{n} I\{A_i=a\}H_{a,i}+o_p(1),$$

where $\Gamma_a=\mathbb{E}[-\partial\mathcal{S}_{a,n}(\beta_a^*)/\partial\beta_a^{*T}]$ is the Fisher information matrix of β_a^*, $H_{a,i}=\int I\{A_i=a\}\{X_i-e_a(\beta_a^*,u)\}\mathrm{d}M_{a,i}(u)$ and $\mathrm{d}M_{a,i}(u)=\mathrm{d}N_i(u)-\exp(\beta_a^{*T}X_i)Y_i(u)\mathrm{d}\Lambda_{0,a}^*(u)$. Moreover, let $S^*(t\,|\,a,X)=\exp\{-\Lambda_{0,a}^*(t)\exp(\beta_a^{*T}X)\}$; it is shown that $\sqrt{n_a}\{\hat S(t\,|\,a,X_i)-S^*(t\,|\,a,X_i)\}$ converges uniformly to a mean-zero Gaussian process for all X_i.

Specifically, we consider the following expansion that we use in our proof of Theorem 6 and Corollary 8,

$$\begin{aligned}\hat S(t\,|\,a,X_i)-S^*(t\,|\,a,X_i)=&-S^*(t\,|\,a,X_i)\Lambda_{0,a}^*(t)\exp(\beta_a^{*T}X_i)X_i^T(\hat\beta_a-\beta_a^*)\\&-S^*(t\,|\,a,X_i)\exp(\beta_a^{*T}X_i)(\hat\Lambda_{0,a}(t)-\Lambda_{0,a}^*(t)),\end{aligned}$$

and furthermore

$$\begin{aligned}
\hat{\Lambda}_{0,a}(t) - \Lambda^*_{0,a}(t) &= \int_0^t \left\{ \frac{n_a^{-1}\sum_{i=1}^n I\{A_i = a\}\mathrm{d}N_i(u)}{U_a^{(0)}(\hat{\beta}_a, u)} - \frac{n_a^{-1}\sum_{i=1}^n I\{A_i = a\}\mathrm{d}N_i(u)}{U_a^{(0)}(\beta_a^*, u)} \right\} \\
&\quad + \int_0^t \left\{ \frac{n_a^{-1}\sum_{i=1}^n I\{A_i = a\}\mathrm{d}N_i(u)}{U_a^{(0)}(\beta_a^*, u)} - \mathrm{d}\Lambda^*_{0,a}(t) \right\} \\
&= -\left[\int_0^t \frac{U_a^{(1)}(\beta_a^*, u)}{\left\{U_a^{(0)}(\beta_a^*, u)\right\}^2} \left\{ n_a^{-1} \sum_{i=1}^n I\{A_i = a\}\mathrm{d}N_i(u) \right\} \right]^T \left(\hat{\beta}_a - \beta_a^*\right) \\
&\quad + \int_0^t \frac{n_a^{-1}\sum_{i=1}^n I\{A_i = a\}\mathrm{d}M_{a,i}(u)}{U_a^{(0)}(\beta_a^*, u)} + o_p(1) \\
&= -\left\{ \int_0^t e_a(\beta_a^*, u)\mathrm{d}\Lambda^*_{0,a}(u) \right\}^T \left(\hat{\beta}_a - \beta_a^*\right) \\
&\quad + \int_0^t \frac{n_a^{-1}\sum_{i=1}^n I\{A_i = a\}\mathrm{d}M_{a,i}(u)}{U_a^{(0)}(\beta_a^*, u)} + o_p(1).
\end{aligned}$$

Combining the above two equations, we obtain

$$\begin{aligned}
&\hat{S}(t \mid a, X_i) - S^*(t \mid a, X_i) \\
&= \left[-S^*(t \mid a, X_i)\Lambda^*_{0,a}(t)\exp(\beta_a^{*T} X_i) X_i^T - \left\{ \int_0^t e_a(\beta_a^*, u)\mathrm{d}\Lambda^*_{0,a}(u) \right\}^T \right] \left(\hat{\beta}_a - \beta_a^*\right) \\
&\quad + \int_0^t \frac{n_a^{-1}\sum_{i=1}^n I\{A_i = a\}\mathrm{d}M_{a,i}(u)}{U_a^{(0)}(\beta_a^*, u)} + o_p(1).
\end{aligned}$$

A.2 Cross-Fitting

To show the high-level idea of cross-fitting, we state the lemma from Kennedy et al. (2020), which is useful in our proof of Theorem 7 and Corollary 9.

Lemma 13 *Consider two independent samples $\mathcal{O}_1 = (O_1, \ldots, O_n)$ and $\mathcal{O}_2 = (O_{n+1}, \ldots, O_{\tilde{n}})$, let $\hat{f}(o)$ be a function estimated from $\mathcal{O}_2$ and $\mathbb{P}_n$ the empirical measure over $\mathcal{O}_1$, then we have*

$$(\mathbb{P}_n - \mathbb{P})(\hat{f} - f) = O_{\mathbb{P}}\left(\frac{\|\hat{f} - f\|}{\sqrt{n}} \right)$$

Proof First note that by conditioning on $\mathcal{O}_2$ we obtain

$$\mathbb{E}\left\{ \mathbb{P}_n(\hat{f} - f) \,\middle|\, \mathcal{O}_2 \right\} = \mathbb{E}(\hat{f} - f \mid \mathcal{O}_2) = \mathbb{P}(\hat{f} - f)$$

and the conditional variance is

$$var\{(\mathbb{P}_n - \mathbb{P})(\hat{f} - f) \mid \mathcal{O}_2\} = var\{\mathbb{P}_n(\hat{f} - f) \mid \mathcal{O}_2\} = \frac{1}{n} var(\hat{f} - f \mid \mathcal{O}_2) \leq \|\hat{f} - f\|^2/n$$

therefore by Chebyshev's inequality we have

$$\mathbb{P}\left\{\frac{|(\mathbb{P}_n-\mathbb{P})(\hat{f}-f)|}{\|\hat{f}-f\|^2/n}\geq t\right\}=\mathbb{E}\left[\mathbb{P}\left\{\frac{|(\mathbb{P}_n-\mathbb{P})(\hat{f}-f)|}{\|\hat{f}-f\|^2/n}\geq t\,\middle|\,\mathcal{O}_2\right\}\right]\leq\frac{1}{t^2}$$

thus for any $\epsilon>0$ we can pick $t=1/\sqrt{\epsilon}$ so that the probability above is no more than ϵ, which yields the result. ■

Appendix B. Proof of Proposition 3

Proof We first show the identification by the outcome regression formula.

$$\begin{aligned}
V(d) &= \mathbb{E}[\mathbb{E}[y(T(d))\mid X]]\\
&= \mathbb{E}[d(X)\mathbb{E}[y(T(1))\mid X]+(1-d(X))\mathbb{E}[y(T(0))\mid X]]\\
&= \mathbb{E}[d(X)\mathbb{E}[y(T(1))\mid X,I_S=1]+(1-d(X))\mathbb{E}[y(T(0))\mid X,I_S=1]]\\
&= \mathbb{E}[d(X)\mathbb{E}[y(T(1))\mid A=1,X,I_S=1]\\
&\qquad +(1-d(X))\mathbb{E}[y(T(0))\mid A=0,X,I_S=1]]\\
&= \mathbb{E}[d(X)\mathbb{E}[y(T)\mid A=1,X,I_S=1]+(1-d(X))\mathbb{E}[y(T)\mid A=0,X,I_S=1]]\\
&= \mathbb{E}[\mathbb{E}[y(T)\mid A=d(X),X,I_S=1]]\\
&= \mathbb{E}[I_T\,e(X)\mathbb{E}[y(T)\mid A=d(X),X,I_S=1]].
\end{aligned}$$

Similarly, we show the identification by the IPW formula.

$$\begin{aligned}
V(d) &= \mathbb{E}[\mathbb{E}[y(T)\mid A=d(X),X,I_S=1]]\\
&= \mathbb{E}\left[\frac{I_S}{\pi_S(X)}\mathbb{E}[y(T)\mid A=d(X),X,I_S=1]\right]\\
&= \mathbb{E}\left[\frac{I_S}{\pi_S(X)}\frac{I\{A=d(X)\}}{\pi_d(X)}\frac{\Delta\,y(U)}{S_C(U\mid A,X)}\right],
\end{aligned}$$

where the last equation follows from the standard IPTW-IPCW formula (van der Laan and Robins, 2003). ■

Appendix C. Proof of Proposition 4

Proof While Lee et al. (2022) derived the efficient influence function for the treatment specific survival function, here we derive the EIF for the value function $V(d)=\mathbb{E}[I_T\,e(X)\mu(d(X),X)]$.

First consider the full data $Z=(X,A,T,I_S,I_T)$, and we have the factorization as

$$p(Z)=\{p(X)\pi_S(X)p(A|X,I_S=1)p(T|A,X,I_S=1)\}^{I_S}\{p(X)\}^{I_T}.$$

Since $I_SI_T=0$, the score function is $S(Z)=S(X,A,T,I_S)+I_TS(X)$. Let $V_\epsilon(d)=\mathbb{E}_\epsilon[I_T\,e(X)\mu_\epsilon(d(X),X)]$ denote the parameter of interest evaluated under the law $p_\epsilon(Z)$,

where ϵ indexes a regular parametric submodel such that $p_0(Z)$ is the true data generating law. To establish that $V(d)$ is pathwise differentiable with EIF ϕ_d^F, we need to show that

$$\frac{\partial}{\partial\epsilon}V_\epsilon(d)\bigg|_{\epsilon=0} = \mathbb{E}[\phi_d^F S(Z)].$$

First, we compute

$$\frac{\partial}{\partial\epsilon}V_\epsilon(d)\bigg|_{\epsilon=0} = \mathbb{E}[I_T\, e(X)\mu(d(X),X)S(X)] + \mathbb{E}\left[\frac{\partial}{\partial\epsilon}\mu_\epsilon(d(X),X)\bigg|_{\epsilon=0}\right],$$

and further write the first term on the right hand side as

$$\begin{aligned}\mathbb{E}[I_T\, e(X)\mu(d(X),X)S(X)] &= \mathbb{E}[(I_T\, e(X)\mu(d(X),X) - V(d))S(X)]\\ &= \mathbb{E}[(I_T\, e(X)\mu(d(X),X) - V(d))S(Z)],\end{aligned}$$

and the second term as

$$\begin{aligned}&\mathbb{E}\left[\frac{\partial}{\partial\epsilon}\mu_\epsilon(d(X),X)\bigg|_{\epsilon=0}\right]\\
&= \mathbb{E}\left[d(X)\mathbb{E}[y(T)S(T\mid A,X,I_S)\mid A=1,X,I_S=1]\right.\\
&\qquad \left.+(1-d(X))\mathbb{E}[y(T)S(T\mid A,X,I_S)\mid A=0,X,I_S=1]\right]\\
&= \mathbb{E}\left[d(X)\mathbb{E}[(y(T)-\mu(1,X))S(T\mid A,X,I_S)\mid A=1,X,I_S=1]\right.\\
&\qquad \left.+(1-d(X))\mathbb{E}[(y(T)-\mu(0,X))S(T\mid A,X,I_S)\mid A=0,X,I_S=1]\right]\\
&= \mathbb{E}\left[d(X)\mathbb{E}\left[\frac{I_S\, A}{\pi_S(X)\pi_A(X)}(y(T)-\mu(1,X))S(T\mid A,X,I_S)\bigg| X\right]\right.\\
&\qquad \left.+(1-d(X))\mathbb{E}\left[\frac{I_S\,(1-A)}{\pi_S(X)(1-\pi_A(X))}(y(T)-\mu(0,X))S(T\mid A,X,I_S)\bigg| X\right]\right]\\
&= \mathbb{E}\left[\frac{I_S}{\pi_S(X)}\left(d(X)\frac{A}{\pi_A(X)}(y(T)-\mu(1,X))\right.\right.\\
&\qquad \left.\left.+(1-d(X))\frac{1-A}{1-\pi_A(X)}(y(T)-\mu(0,X))\right)S(T\mid A,X,I_S)\right]\\
&= \mathbb{E}\left[\frac{I_S}{\pi_S(X)}\frac{I\{A=d(X)\}}{\pi_d(X)}(y(T)-\mu(A,X))S(Z)\right].\end{aligned}$$

Therefore, the efficient influence function for the full data is

$$\phi_d^F = I_T\, e(X)\mu(d(X),X) + \frac{I_S}{\pi_S(X)}\frac{I\{A=d(X)\}}{\pi_d(X)}(y(T)-\mu(A,X)) - V(d).$$

Next, we consider the observed data $O = (X, A, U, \Delta, I_S, I_T)$ due to right censoring. According to Tsiatis (2006, Section 10.4), the EIF based on the observed data is given by

$$\phi_d = \frac{\Delta\,\phi_d^F}{S_C(U\mid A,X)} + \int_0^\infty \frac{L(u,A,X)}{S_C(u\mid A,X)}\mathrm{d}M_C(u\mid A,X),$$

where

$$\begin{aligned}
L(u, A, X) &= \mathbb{E}[\phi_d^F \,|\, T \geq u, A, X] \\
&= I_T\, e(X)\mu(d(X), X) + \frac{I_S}{\pi_S(X)} \frac{I\{A = d(X)\}}{\pi_d(X)} (Q(u, A, X) - \mu(A, X)) - V(d).
\end{aligned}$$

Since we have

$$\begin{aligned}
\int_0^\infty \frac{\mathrm{d}M_C(u \,|\, A, X)}{S_C(u \,|\, A, X)} &= \int_0^\infty \frac{\mathrm{d}N_C(u)}{S_C(u \,|\, A, X)} - \int_0^U \frac{\mathrm{d}\Lambda_C(u \,|\, A, X)}{\exp\{\Lambda_C(u \,|\, A, X)\}} \\
&= 1 - \frac{\Delta}{S_C(U \,|\, A, X)},
\end{aligned} \tag{9}$$

we conclude that

$$\begin{aligned}
\phi_d =& \frac{I_S}{\pi_S(X)} \frac{I\{A = d(X)\}}{\pi_d(X)} \frac{\Delta\, y(U)}{S_C(U \,|\, A, X)} - V(d) \\
&+ \left(I_T\, e(X) - \frac{I_S}{\pi_S(X)} \frac{I\{A = d(X)\}}{\pi_d(X)} \right) \mu(d(X), X) \\
&+ \frac{I_S}{\pi_S(X)} \frac{I\{A = d(X)\}}{\pi_d(X)} \int_0^\infty \frac{\mathrm{d}M_C(u \,|\, A, X)}{S_C(u \,|\, A, X)} Q(u, A, X).
\end{aligned}$$

■

Appendix D. Proof of Theorem 6 and Corollary 8

D.1 Double Robustness

Proof We start with the proof of the double robustness property. We show that EIF-based estimator is consistent when either the survival outcome model or the models for the sampling score, the propensity score and the censoring process are correctly specified. Under some regularity conditions, the nuisance estimators $\hat{\mu}(a, x)$, $\hat{Q}(u, a, x)$, $\hat{\pi}_S(x)$, $\hat{\pi}_A(x)$ and $\hat{S}_C(t \,|\, a, x)$ converge in probability to $\mu^*(a, x)$, $Q^*(u, a, x)$, $\pi_S^*(x)$, $\pi_A^*(x)$ and $S_C^*(t \,|\, a, x)$, respectively. It suffices to show that $\mathbb{E}[V^*(d)] = V(d)$, where

$$\begin{aligned}
V^*(d) =& I_T\, e(X)\mu^*(A = d(X), X) \\
&+ \frac{I_S}{\pi_S^*(X)} \frac{I\{A = d(X)\}}{\pi_d^*(X)} \left\{ \frac{\Delta\, y(U)}{S_C^*(U \,|\, A, X)} - \mu^*(A, X) \right. \\
&\left. + \int_0^\infty \frac{\mathrm{d}M_C^*(u \,|\, A, X)}{S_C^*(u \,|\, A, X)} Q^*(u, A, X) \right\} \\
=& (I) + (II) + (III).
\end{aligned}$$

First, consider the case when the survival outcome model is correct, thus we have

$$(I) = \mathbb{E}[I_T\, e(X)\mu^*(A = d(X), X)] = V(d)$$

By Equation (9), we obtain

$$
\begin{aligned}
&(II)+(III)\\
&=\frac{I_S}{\pi_S^*(X)}\frac{I\{A=d(X)\}}{\pi_d^*(X)}\left\{y(T)-\mu^*(A,X)-\int_0^\infty \frac{\mathrm{d}M_C^*(u\,|\,A,X)}{S_C^*(u\,|\,A,X)}(y(T)-Q^*(u,A,X))\right\}.
\end{aligned}
$$

In this case, we have

$$
\begin{aligned}
&\mathbb{E}\left[\frac{I_S}{\pi_S^*(X)}\frac{I\{A=d(X)\}}{\pi_d^*(X)}(y(T)-\mu^*(A,X))\right]\\
&=\mathbb{E}\left[\mathbb{E}\left[\frac{I_S}{\pi_S^*(X)}\frac{I\{A=d(X)\}}{\pi_d^*(X)}(y(T)-\mu^*(A,X))\,\middle|\,X\right]\right]\\
&=\mathbb{E}\left[\mathbb{E}\left[\mathbb{E}\left[\frac{I_S}{\pi_S^*(X)}\frac{I\{A=d(X)\}}{\pi_d^*(X)}(y(T)-\mu^*(A,X))\,\middle|\,A,X,I_S=1\right]\,\middle|\,X\right]\right]\\
&=\mathbb{E}\left[\mathbb{E}\left[\frac{I_S}{\pi_S^*(X)}\frac{I\{A=d(X)\}}{\pi_d^*(X)}\mathbb{E}[(y(T)-\mu^*(A,X))\,|\,A,X,I_S=1]\,\middle|\,X\right]\right]\\
&=\mathbb{E}\left[\mathbb{E}\left[\frac{I_S}{\pi_S^*(X)}\frac{I\{A=d(X)\}}{\pi_d^*(X)}(\mathbb{E}[y(T)\,|\,A,X,I_S=1]-\mu^*(A,X))\,\middle|\,X\right]\right]=0.
\end{aligned}
$$

Also define $\mathrm{d}\tilde{M}_C(u\,|\,A,X)=\mathrm{d}\tilde{N}_C(u)-I\{C\geq u\}\mathrm{d}\Lambda_C(u\,|\,A,X)$ where $\tilde{N}_C(u)=I\{C\leq u\}$, so we have

$$
\begin{aligned}
&\mathbb{E}\left[\frac{I_S}{\pi_S^*(X)}\frac{I\{A=d(X)\}}{\pi_d^*(X)}\int_0^\infty \frac{\mathrm{d}M_C^*(u\,|\,A,X)}{S_C^*(u\,|\,A,X)}(y(T)-Q^*(u,A,X))\right]\\
&=\mathbb{E}\left[\frac{I_S}{\pi_S^*(X)}\frac{I\{A=d(X)\}}{\pi_d^*(X)}\int_0^\infty \frac{\mathrm{d}\tilde{M}_C(u\,|\,A,X)}{S_C^*(u\,|\,A,X)}I\{T\geq u\}(y(T)-Q^*(u,A,X))\right]\\
&=\mathbb{E}\left[\mathbb{E}\left[\frac{I_S}{\pi_S^*(X)}\frac{I\{A=d(X)\}}{\pi_d^*(X)}\int_0^\infty \frac{\mathrm{d}\tilde{M}^{(}_C u\,|\,A,X)}{S_C^*(u\,|\,A,X)}I\{T\geq u\}(y(T)-Q^*(u,A,X))\,\middle|\,X\right]\right]\\
&=\mathbb{E}\left[\mathbb{E}\left[\mathbb{E}\left[\frac{I_S}{\pi_S^*(X)}\frac{I\{A=d(X)\}}{\pi_d^*(X)}\int_0^\infty \frac{\mathrm{d}\tilde{M}_C(u\,|\,A,X)}{S_C^*(u\,|\,A,X)}I\{T\geq u\}\right.\right.\right.\\
&\qquad\left.\left.\left.(y(T)-Q^*(u,A,X))\,\middle|\,A,X,C,I_S=1\right]\,\middle|\,X\right]\right]\\
&=\mathbb{E}\left[\mathbb{E}\left[\frac{I_S}{\pi_S^*(X)}\frac{I\{A=d(X)\}}{\pi_d^*(X)}\int_0^\infty \frac{\mathrm{d}\tilde{M}_C(u\,|\,A,X)}{S_C^*(u\,|\,A,X)}\mathbb{E}\left[I\{T\geq u\}\right.\right.\right.\\
&\qquad\left.\left.\left.(y(T)-Q^*(u,A,X))\,\middle|\,A,X,C,I_S=1\right]\,\middle|\,X\right]\right]\\
&=\mathbb{E}\left[\mathbb{E}\left[\frac{I_S}{\pi_S^*(X)}\frac{I\{A=d(X)\}}{\pi_d^*(X)}\int_0^\infty \frac{\mathrm{d}\tilde{M}_C(u\,|\,A,X)}{S_C^*(u\,|\,A,X)}\left(\mathbb{E}[I\{T\geq u\}y(T)\,|\,A,X,I_S=1]\right.\right.\right.\\
&\qquad\left.\left.\left.-\mathbb{E}[I\{T\geq u\}\,|\,A,X,I_S=1]Q^*(u,A,X)\right)\,\middle|\,X\right]\right]=0.
\end{aligned}
$$

Next, consider the case when the models for the sampling score, the propensity score and the censoring process are correctly specified. Rearranging the terms of $V^*(d)$, we obtain

$$\begin{aligned}V^*(d) =&\frac{I_S}{\pi_S^*(X)}\frac{I\{A=d(X)\}}{\pi_d^*(X)}\frac{\Delta\, y(U)}{S_C^*(U\,|\,A,X)}\\&+\left(I_T\, e(X)-\frac{I_S}{\pi_S^*(X)}\right)\mu^*(A=d(X),X)\\&+\frac{I_S}{\pi_S^*(X)}\frac{I\{A=d(X)\}}{\pi_d^*(X)}\int_0^\infty\frac{\mathrm{d}M_C^*(u\,|\,A,X)}{S_C^*(u\,|\,A,X)}Q^*(u,A,X)\\=&(I)+(II)+(III).\end{aligned}$$

In this case, we have

$$\begin{aligned}(I)&=\mathbb{E}\left[\frac{I_S}{\pi_S^*(X)}\frac{I\{A=d(X)\}}{\pi_d^*(X)}\frac{\Delta\, y(U)}{S_C^*(U\,|\,A,X)}\right]=V(d),\\(II)&=\mathbb{E}\left[\left(I_T\, e(X)-\frac{I_S}{\pi_S^*(X)}\right)\mu^*(A=d(X),X)\right]\\&=\mathbb{E}\left[\mathbb{E}\left[I_T\, e(X)-\frac{I_S}{\pi_S^*(X)}\middle|X\right]\mu^*(A=d(X),X)\right]=0,\end{aligned}$$

and (III) is a stochastic integral with respect to the martingale $M_C^*(u\,|\,A,X)$, thus equals 0 as well, which completes the double robustness property. ■

D.2 Asymptotic Properties

To establish the asymptotic results, we need some regularity conditions such that the nuisance estimators $\mu(a,x;\hat\beta_a,\hat\Lambda_{0,a})$, $Q(u,a,x;\hat\beta_a,\hat\Lambda_{0,a})$, $\pi_S(x;\hat\lambda)$, $\pi_A(x;\hat\theta)$ and $S_C(u\,|\,a,x;\hat\alpha_a,\hat\Lambda_{C0,a})$ converge in probability to $\mu(a,x;\beta_a^*,\Lambda_{0,a}^*)$, $Q(u,a,x;\beta_a^*,\Lambda_{0,a}^*)$, $\pi_S(x;\lambda^*)$, $\pi_A(x;\theta^*)$ and $S_C(t\,|\,a,x;\alpha_a^*,\Lambda_{C0,a}^*)$, respectively.

Condition 1 *We assume the following conditions hold:*
(C1) X is bounded almost surely.
(C2) The equation $\mathbb{E}\left[\left\{A-\frac{\exp(\theta^TX)}{1+\exp(\theta^TX)}\right\}X\right]=0$ has a unique solution θ^*.
(C3) For $a=0,1$, the equation

$$\mathbb{E}\left[\int_0^L\left(X_i-\frac{\mathbb{E}[Y_i(u)\exp(\beta_a^TX)X]}{\mathbb{E}[Y_i(u)\exp(\beta_a^TX)]}\right)\times\mathrm{d}N_i(u)\right]=0,$$

has a unique solution β_a^*, where $L>u$ is a pre-specified time point such that $Pr(U_i>L)>0$. Moreover, let

$$\Lambda_{0,a}^*(u)=\mathbb{E}\left[\int_0^u\frac{\mathrm{d}N_i(u)}{\mathbb{E}[Y_i(u)\exp(\beta_a^{*T}X_i)]}\right],$$

and assume $\Lambda_{0,a}^*(L)<\infty$.
(C4) For $a=0,1$, the equation

$$\mathbb{E}\left[\int_0^L\left(X_i-\frac{\mathbb{E}[Y_i(u)\exp(\alpha_a^TX)X]}{\mathbb{E}[Y_i(u)\exp(\alpha_a^TX)]}\right)\times\mathrm{d}N_i(u)\right]=0,$$

has a unique solution α_a^*. Moreover, let

$$\Lambda_{C0,a}^*(u) = \mathbb{E}\left[\int_0^u \frac{\mathrm{d}N_i(u)}{\mathbb{E}[Y_i(u)\exp(\alpha_a^{*T} X_i)]}\right],$$

and assume $\Lambda_{C0,a}^*(L) < \infty$.
(C5) The estimating equation for the sampling score model $\pi_S(X;\lambda)$ has a unique solution λ^*, and achieves root-n rate of convergence.

Under Condition 1, we have the following asymptotic representations:

$$\sqrt{n}(\hat\theta - \theta^*) = \frac{1}{\sqrt{n}}\sum_{i=1}^n \phi_{\theta i} + o_p(1), \qquad \sqrt{n}(\hat\lambda - \lambda^*) = \frac{1}{\sqrt{n}}\sum_{i=1}^n \phi_{\lambda i} + o_p(1),$$

$$\sqrt{n}(\hat\beta_a - \beta_a^*) = \frac{1}{\sqrt{n}}\sum_{i=1}^n \phi_{\beta_a i} + o_p(1), \quad \sqrt{n}(\hat\alpha_a - \alpha_a^*) = \frac{1}{\sqrt{n}}\sum_{i=1}^n \phi_{\alpha_a i} + o_p(1), \quad \text{for } a = 0, 1.$$

For the ease of notation, we introduce a finite population of size N, from which the source and target data are independently sampled. Note that we do not assume N is known and fixed.

We focus on the estimation of survival functions by our proposed method:

$$\begin{aligned}\hat S(t;\eta) =& \frac{1}{N}\sum_{i=1}^N \Bigg[I_{T,i}\, e(X_i)\hat S(t \mid A = d_\eta(X_i), X_i) \\ &+ \frac{I_{S,i} I\{A_i = d_\eta(X_i)\}}{\hat\pi_S(X_i)\hat\pi_d(X_i)} \Bigg\{ \frac{\Delta_i\, Y_i(t)}{\hat S_C(t \mid A_i, X_i)} - \hat S(t \mid A_i, X_i) \\ &+ \int_0^\infty \frac{\hat S(t \mid A_i, X_i)\mathrm{d}\hat M_C(u \mid A_i, X_i)}{\hat S(u \mid A_i, X_i)\hat S_C(u \mid A_i, X_i)} \Bigg\} \Bigg],\end{aligned}$$

and for the ease of notation, define

$$\hat J(t,a,x) = \frac{\Delta_i\, Y_i(t)}{\hat S_C(t \mid a, x)} - \hat S(t \mid a, x) + \int_0^\infty \frac{\hat S(t \mid a, x)\mathrm{d}\hat M_C(u \mid a, x)}{\hat S(u \mid a, x)\hat S_C(u \mid a, x)},$$

$$J^*(t,a,x) = \frac{\Delta_i\, Y_i(t)}{S_C^*(t \mid a, x)} - S^*(t \mid a, x) + \int_0^\infty \frac{S^*(t \mid a, x)\mathrm{d}M_C^*(u \mid a, x)}{S^*(u \mid a, x)S_C^*(u \mid a, x)}.$$

Proof Our proof has three main parts as follows.

PART 1. By the double robustness property shown in Section D.1, we have, by the strong law of large numbers and uniform consistency, that $\hat S(t;\eta) = S(t;\eta) + o_p(1)$, which proves (i) of Theorem 6. Moreover, define

$$S_N^*(t;\eta) = \frac{1}{N}\sum_{i=1}^N \left[I_{T,i}\, e(X_i)S^*(t \mid A = d_\eta(X_i), X_i) + \frac{I_{S,i} I\{A_i = d_\eta(X_i)\}}{\pi_S^*(X_i)\pi_d^*(X_i)} J^*(t, A_i, X_i) \right],$$

and by applying the Taylor expansion and the counting processes result in Section A.1, we obtain

$$\hat{S}(t;\eta) = S_n^*(t;\eta) + H_\lambda^T(\hat{\lambda}-\lambda^*) + H_\theta^T(\hat{\theta}-\theta^*) + H_{\beta_0}^T(\hat{\beta}_0-\beta_0^*) + H_{\beta_1}^T(\hat{\beta}_1-\beta_1^*) + H_{\alpha_0}^T(\hat{\alpha}_0-\alpha_0^*) + H_{\alpha_1}^T(\hat{\alpha}_1-\alpha_1^*) + R_S + o_p(N^{-1/2}),$$

where

$$H_\lambda = \lim_{N\to\infty}\frac{1}{N}\sum_{i=1}^N \frac{\partial \hat{S}(t;\eta)}{\partial \lambda^*}, H_\theta = \lim_{N\to\infty}\frac{1}{N}\sum_{i=1}^N \frac{\partial \hat{S}(t;\eta)}{\partial \theta^*},$$

$$H_{\beta_a} = \lim_{N\to\infty}\frac{1}{N}\sum_{i=1}^N \Bigg\{ I_{T,i}\, e(X_i)(-1)^{a+1}G(t,a,X_i) + \frac{I_{S,i}I\{A_i=a\}}{\pi_S^*(X_i)\pi_d^*(X_i)}\Bigg(\int_0^\infty \frac{G(t,a,X_i)\mathrm{d}M_C^*(u\,|\,a,X_i)}{S^*(u\,|\,a,X_i)S_C^*(u\,|\,a,X_i)} - G(t,a,X_i) - \int_0^\infty \frac{G(u,a,X_i)S^*(t\,|\,a,X_i)\mathrm{d}M_C^*(u\,|\,a,X_i)}{S^{*2}(u\,|\,a,X_i)S_C^*(u\,|\,a,X_i)}\Bigg)\Bigg\},$$

$$H_{\alpha_a} = \lim_{N\to\infty}\frac{1}{N}\sum_{i=1}^N \frac{I_{S,i}I\{A_i=a\}}{\pi_S^*(X_i)\pi_d^*(X_i)}\Bigg\{\frac{-\Delta_i Y_i(t)}{S_C^*(t\,|\,a,X_i)}G_C(t,a,X_i) - \int_0^\infty \frac{G_C(u,a,X_i)S^*(t\,|\,a,X_i)\mathrm{d}M_C^*(u\,|\,a,X_i)}{S_C^{*2}(u\,|\,a,X_i)S^*(u\,|\,a,X_i)} + \tilde{G}_C(t,a,X_i)\Bigg\},$$

$$\begin{aligned} R_S = \frac{1}{N}\sum_{i=1}^N\sum_{a=0,1}\Bigg\{ & I_{T,i}\, e(X_i)(-1)^{a+1}H(t,a,X_i) \\ & + \frac{I_{S,i}I\{A_i=a\}}{\pi_S^*(X_i)\pi_d^*(X_i)}\Bigg(\int_0^\infty \frac{H(t,a,X_i)\mathrm{d}M_C^*(u\,|\,a,X_i)}{S_C^*(u\,|\,a,X_i)S^*(u\,|\,a,X_i)} - H(t,a,X_i) \\ & - \int_0^\infty \frac{H(u,a,X_i)S^*(t\,|\,a,X_i)\mathrm{d}M_C^*(u\,|\,a,X_i)}{S_C^*(u\,|\,a,X_i)S^{*2}(u\,|\,a,X_i)} - \frac{\Delta_i Y_i(t)}{S_C^*(t|a,X_i)}H_C(t,a,X_i) \\ & - \int_0^\infty \frac{H_C(u,a,X_i)S^*(t\,|\,a,X_i)\mathrm{d}M_C^*(u\,|\,a,X_i)}{S_C^{*2}(u\,|\,a,X_i)S^*(u\,|\,a,X_i)} - \tilde{H}_C(t,a,X_i)\Bigg)\Bigg\} \\ = \frac{1}{N}\sum_{i=1}^N \phi_{Rs,i}, \end{aligned}$$

with

$$G(t,a,x) = -S^*(t\,|\,a,x)\Lambda_{0,a}^*(t)x^T + S^*(t\,|\,a,x)\exp(\beta_a^{*T}x)\left\{\int_0^t e_a(\beta_a^*,u)\mathrm{d}\Lambda_{0,a}^*(u)\right\}^T,$$

$$H(t,a,x) = -S^*(t\,|\,a,x)\exp(\beta_a^{*T}x)\int_0^t \frac{n_a^{-1}\sum_{i=1}^n I\{A_i=a\}\mathrm{d}M_{a,i}(u)}{U_a^{(0)}(\beta_a^*,u)},$$

$$G_C(t,a,x) = -S^*(t\,|\,a,x)\Lambda_{0,a}^*(t)x^T + S^*(t\,|\,a,x)\exp(\beta_a^{*T}x)\left\{\int_0^t e_a(\beta_a^*,u)\mathrm{d}\Lambda_{0,a}^*(u)\right\}^T,$$

$$H_C(t,a,x) = -S^*(t\,|\,a,x)\exp(\beta_a^{*T}x)\int_0^t \frac{n_a^{-1}\sum_{i=1}^n I\{A_i=a\}\mathrm{d}M_{a,i}(u)}{U_a^{(0)}(\beta_a^*,u)},$$

$$\tilde{G}_C(t,a,x) = \int_0^{U_i} \frac{S^*(t\,|\,a,x)\mathrm{d}\Lambda_C^*(u\,|\,a,x)}{S_C^*(u\,|\,a,x)S^*(u\,|\,a,x)} x^T + \left\{\int_0^t \frac{S^*(t\,|\,a,x)e_a(\beta_a^*,u)\mathrm{d}\Lambda_{0,a}^*(u)}{S_C^*(u\,|\,a,x)S^*(u\,|\,a,x)}\right\}^T,$$

$$\tilde{H}_C(t,a,x) = \int_0^t \frac{S^*(t\,|\,a,x)n_a^{-1}\sum_{i=1}^n I\{A_i=a\}\mathrm{d}M_{a,i}(u)}{S_C^*(u\,|\,a,x)S^*(u\,|\,a,x)U_a^{(0)}(\beta_a^*,u)}.$$

Thus, we have

$$\sqrt{N}\left\{\hat{S}(t;\eta) - S(t;\eta)\right\} = \frac{1}{\sqrt{N}}\sum_{i=1}^N (\xi_{1,i}(t;\eta) + \xi_{2,i}(t;\eta)) + o_p(1), \tag{10}$$

where

$$\xi_{1,i}(t;\eta) = S_n^*(t;\eta) - S(t;\eta),$$

$$\xi_{2,i}(t;\eta) = H_\lambda^T\phi_{\lambda^*,i} + H_\theta^T\phi_{\theta^*,i} + \sum_{a=0,1} H_{\beta_a}^T\phi_{\beta_0^*,i} + \sum_{a=0,1} H_{\alpha_a}^T\phi_{\alpha_a^*,i} + H_{\alpha_1}^T + \phi_{Rs,i},$$

and $\xi_{1,i}(t;\eta), \xi_{2,i}(t;\eta)$ are independent mean-zero processes. Therefore, we obtain that $\sqrt{N}\left\{\hat{S}(t;\eta) - S(t;\eta)\right\}$ converges weakly to a mean-zero Gaussian process, which proves (ii) of Theorem 6.

PART 2. We show that $N^{1/3}\|\hat{\eta} - \eta^*\|_2 = O_p(1)$. Recall that

$$\hat{\eta} = \arg\max_\eta \hat{S}(t;\eta) \text{ and } \eta^* = \arg\max_\eta S(t;\eta).$$

By Assumption 5 (i), $S(t;\eta)$ is twice continuously differentiable at a neighborhood of η^*; in Step 1, we show that $\hat{S}(t;\eta) = S(t;\eta) + o_p(1), \forall\eta$; since $\hat{\eta}$ maximizes $\hat{S}(t;\eta)$, we have that $\hat{S}(t;\hat{\eta}) \geq \sup_\eta \hat{S}(t;\eta)$, thus by the Argmax theorem, we have $\hat{\eta} \xrightarrow{p} \eta^*$ as $N \to \infty$.

In order to establish the $N^{-1/3}$ rate of convergence of $\hat{\eta}$, we apply Theorem 14.4 (Rate of convergence) of Kosorok (2008), and need to find the suitable rate that satisfies three conditions below.

Condition 1 For every η in a neighborhood of η^* such that $\|\eta - \eta^*\|_2 < \delta$, by Assumption 5 (i), we apply the second-order Taylor expansion,

$$\begin{aligned} S(t;\eta) - S(t;\eta^*) &= S'(\eta^*)\|\eta - \eta^*\|_2 + \frac{1}{2}S''(\eta^*)\|\eta - \eta^*\|_2^2 + o(\|\eta - \eta^*\|_2^2) \\ &= \frac{1}{2}S''(\eta^*)\|\eta - \eta^*\|_2^2 + o(\|\eta - \eta^*\|_2^2), \end{aligned}$$

and as $S''(\eta^*) < 0$, there exists $c_0 = -\frac{1}{2}S''(\eta^*) > 0$ such that $S(t;\eta) - S(t;\eta^*) \leq -c_0\|\eta - \eta^*\|_2^2$.

Condition 2 For all N large enough and sufficiently small δ, we consider the centered process $\hat{S} - S$, and have that

$$\begin{aligned}
&\mathbb{E}\left[\sqrt{N} \sup_{\|\eta-\eta^*\|_2<\delta} \left|\hat{S}(t;\eta) - S(t;\eta) - \left\{\hat{S}(t;\eta^*) - S(t;\eta^*)\right\}\right|\right] \\
&= \mathbb{E}\Bigg[\sqrt{N} \sup_{\|\eta-\eta^*\|_2<\delta} \Big|\hat{S}(t;\eta) - S_n^*(t;\eta) + S_n^*(t;\eta) - S(t;\eta) \\
&\qquad - \left\{\hat{S}(t;\eta^*) - S_n^*(t;\eta^*) + S_n^*(t;\eta^*) - S(t;\eta^*)\right\}\Big|\Bigg] \\
&\leq \mathbb{E}\left[\sqrt{N} \sup_{\|\eta-\eta^*\|_2<\delta} \left|\hat{S}(t;\eta) - S_n^*(t;\eta) - \left\{\hat{S}(t;\eta^*) - S_n^*(t;\eta^*)\right\}\right|\right] \qquad (I) \\
&\quad + \mathbb{E}\left[\sqrt{N} \sup_{\|\eta-\eta^*\|_2<\delta} \left|S_n^*(t;\eta) - S(t;\eta) - \{S_n^*(t;\eta^*) - S(t;\eta^*)\}\right|\right], \qquad (II)
\end{aligned}$$

and we bound (I) and (II) respectively as follows.

Condition 2.1 To bound (II), we need the useful facts that

$$I\{A = d_\eta(X)\} - I\{A = d_{\eta*}(X)\} = (2A-1)(d_\eta(X) - d_{\eta*}(X)),$$

$$S^*(t \,|\, d_\eta(X_i), X_i) - S^*(t \,|\, d_{\eta^*}(X_i), X_i) = (S^*(t \,|\, 1, X_i) - S^*(t \,|\, 0, X_i))(d_\eta(X_i) - d_{\eta*}(X_i)),$$

and obtain

$$\begin{aligned}
S_n^*(t;\eta) - S_n^*(t;\eta^*) &= \frac{1}{N}\sum_{i=1}^{N}(d_\eta(X_i) - d_{\eta*}(X_i)) \\
&\times \left\{I_{T,i}\, e(X_i)(S^*(t \,|\, 1, X_i) - S^*(t \,|\, 0, X_i)) + \frac{(2A_i-1)I_{S,i}}{\pi_S^*(X_i)\pi_d^*(X_i)} J^*(t, A_i, X_i)\right\}.
\end{aligned}$$

Define a class of functions

$$\begin{aligned}
\mathcal{F}_\eta^1 = &\Bigg\{(d_\eta(x) - d_{\eta*}(x))\left(I_T\, e(x)(S^*(t \,|\, 1, x) - S^*(t \,|\, 0, x)) + \frac{(2a-1)I_S}{\pi_a^*(x)\pi_S^*(x)} J^*(t, a, x)\right) : \\
&\qquad \|\eta - \eta^*\|_2 < \delta\Bigg\},
\end{aligned}$$

and let $M_1 = \sup\left|I_T\, e(x)(S^*(t \,|\, 1, x) - S^*(t \,|\, 0, x)) + \frac{(2a-1)I_S}{\pi_a^*(x)\pi_S^*(x)} J^*(t, a, x)\right|$. By Assumption 1, 3 and Condition 1, we have that $M_1 < \infty$.

When $\|\eta - \eta^*\|_2 < \delta$, by Condition 1 *(C1)*, there exists a constant $0 < k_0 < \infty$ such that $|(1, x^T)(\eta - \eta^*)| < k_0\delta$; furthermore, we show that $|d_\eta(x) - d_{\eta*}(x)| = |I\{(1, x^T)\eta > 0\} - I\{(1, x^T)\eta^* > 0\}| \leq I\{-k_0\delta \leq (1, x^T)\eta^* \leq k_0\delta\}$, by considering the three cases:

- when $-k_0\delta \leq (1, x^T)\eta^* \leq k_0\delta$, we have $|d_\eta(x) - d_{\eta*}(x)| \leq 1 = I\{-k_0\delta \leq (1, x^T)\eta^* \leq k_0\delta\}$;

- when $(1, x^T)\eta^* > k_0\delta > 0$, we have $(1, x^T)\eta = (1, x^T)(\eta - \eta^*) + (1, x^T)\eta^* > 0$, so $|d_\eta(x) - d_{\eta*}(x)| = 0 = I\{-k_0\delta \le (1, x^T)\eta^* \le k_0\delta\}$;
- when $(1, x^T)\eta^* < -k_0\delta < 0$, we have $(1, x^T)\eta = (1, x^T)(\eta - \eta^*) + (1, x^T)\eta^* < 0$, so $|d_\eta(x) - d_{\eta*}(x)| = 0 = I\{-k_0\delta \le (1, x^T)\eta^* \le k_0\delta\}$.

Thus we can define the envelope of $\mathcal{F}_\eta^1$ as $F_1 = M_1 I\{-k_0\delta \le (1, x^T)\eta^* \le k_0\delta\}$. By Assumption 5 *(ii)*, there exists a constant $0 < k_1 < \infty$ such that

$$\|F_1\|_{P,2} \le M_1\sqrt{Pr(-k_0\delta \le (1, x^T)\eta^* \le k_0\delta)} \le M_1\sqrt{2k_0k_1}\delta^{1/2}.$$

By Lemma 9.6 and Lemma 9.9 of Kosorok (2008), we have that $\mathcal{F}_\eta^1$, a class of indicator functions, is a Vapnik-Cervonenkis (VC) class with bounded bracketing entropy $J_{[]}^*(1, \mathcal{F}_\eta^1) < \infty$.

Since we have the fact that

$$\begin{aligned}\mathbb{G}_N\mathcal{F}_\eta^1 &= N^{-1/2}\sum_{i=1}^N \left\{\mathcal{F}_\eta^1 - \mathbb{E}[\mathcal{F}_\eta^1]\right\} \\ &= \sqrt{N}\left(S_n^*(t;\eta) - S_n^*(t;\eta^*) - \{S(t;\eta) - S(t;\eta^*)\}\right),\end{aligned}$$

By Theorem 11.2 of Kosorok (2008), we obtain that there exists a constant $0 < c_1 < \infty$,

$$(II) = \mathbb{E}\left[\sup_{\|\eta-\eta^*\|_2<\delta}|\mathbb{G}_N\mathcal{F}_\eta^1|\right] \le c_1 J_{[]}^*(1, \mathcal{F}_\eta^1)\|F_1\|_{P,2} \le c_1 J_{[]}^*(1, \mathcal{F}_\eta^1)M_1\sqrt{2k_0k_1}\delta^{1/2} = \tilde{c}_1\delta^{1/2},$$

so we conclude that $(II) \le \tilde{c}_1\delta^{1/2}$ where $\tilde{c}_1 > 0$ is a finite constant.

Condition 2.2 To bound (I), first we have

$$\begin{aligned}&\hat{S}(t;\eta) - S_n^*(t;\eta) - \{\hat{S}(t;\eta^*) - S_n^*(t;\eta^*)\} = \hat{S}(t;\eta) - \hat{S}(t;\eta^*) - \{S_n^*(t;\eta) - S_n^*(t;\eta^*)\} \\ &= \frac{1}{N}\sum_{i=1}^N (d_\eta(X_i) - d_{\eta*}(X_i))\Big[I_{T,i}\, e(X_i)\{\hat{S}(t|1, X_i) - \hat{S}(t|0, X_i) - (S^*(t|1, X_i) - S^*(t|0, X_i))\} \\ &\quad + \frac{(2A_i - 1)I_{S,i}}{\hat{\pi}_{A_i}(X_i)\hat{\pi}_S(X_i)}\hat{J}(t, A_i, X_i) - \frac{(2A_i - 1)I_{S,i}}{\pi_{A_i}^*(X_i)\pi_S^*(X_i)}J^*(t, A_i, X_i)\Big],\end{aligned}$$

and then apply the Taylor expansion and counting processes result in Section A.1,

$$\begin{aligned}&\hat{S}(t;\eta) - S_n^*(t;\eta) - \{\hat{S}(t;\eta^*) - S_n^*(t;\eta^*)\} \\ &= \frac{1}{N}\sum_{i=1}^N (d_\eta(X_i) - d_{\eta*}(X_i)) \times \Big\{D_\lambda(\hat{\lambda} - \lambda^*) + D_\theta(\hat{\theta} - \theta^*) + D_{\beta_0}(\hat{\beta}_0 - \beta_0^*) \\ &\quad + D_{\beta_1}(\hat{\beta}_1 - \beta_1^*) + D_{\alpha_0}(\hat{\alpha}_0 - \alpha_0^*) + D_{\alpha_1}(\hat{\alpha}_1 - \alpha_1^*) + R_{S,i}\Big\} + o_p(N^{-1/2}),\end{aligned} \tag{11}$$

where

$$D_\lambda = -\frac{(2A_i - 1)I_{S,i}}{\pi_{A_i}^*(X_i)\pi_S^{*2}(X_i)}J^*(t, A_i, X_i)\left(\frac{\partial\pi_S^*(X_i)}{\partial\lambda}\right)^T,$$

$$D_\theta = -\frac{I_{S,i}}{\pi^{*2}_{A_i}(X_i)\pi^*_S(X_i)} J^*(t, A_i, X_i) \left(\frac{\partial \pi^*_A(X_i)}{\partial \theta}\right)^T,$$

$$\begin{aligned}D_{\beta_a} =& I_{T,i}\, e(X_i)(-1)^{a+1} G(t,a,X_i) + \frac{(2A_i-1)I\{A_i=a\}I_{S,i}}{\pi^*_{A_i}(X_i)\pi^*_S(X_i)}\left(\int_0^\infty \frac{G(t,a,X_i)\mathrm{d}M^*_C(u \mid a, X_i)}{S^*_C(u \mid a, X_i)S^*(u \mid a, X_i)}\right.\\ &\left. - G(t,a,X_i) - \int_0^\infty \frac{G(u,a,X_i)S^*(t \mid a, X_i)\mathrm{d}M^*_C(u \mid a, X_i)}{S^*_C(u \mid a, X_i)S^{*2}(u \mid a, X_i)}\right),\end{aligned}$$

$$\begin{aligned}D_{\alpha_a} =& \frac{(2A_i-1)I\{A_i=a\}I_{S,i}}{\pi^*_{A_i}(X_i)\pi^*_S(X_i)}\left\{ -\frac{\Delta_i\, Y_i(t)}{S^*_C(t \mid a, X_i)} G_C(t,a,X_i)\right.\\ &\left. - \int_0^\infty \frac{G_C(u,a,X_i)S^*(t \mid a, X_i)\mathrm{d}M^*_C(u \mid a, X_i)}{S^{*2}_C(u \mid a, X_i)S^*(u \mid a, X_i)} + \tilde{G}_C(t,a,X_i)\right\},\end{aligned}$$

$$\begin{aligned}R_{S,i} = \sum_{a=0,1} \Bigg[& I_{T,i}\, e(X_i)(-1)^{a+1} H(t,a,X_i) + \frac{(2A_i-1)I\{A_i=a\}I_{S,i}}{\pi^*_{A_i}(X_i)\pi^*_S(X_i)}\Bigg(\int_0^\infty \frac{H(t,a,X_i)\mathrm{d}M^*_C(u \mid a, X_i)}{S^*_C(u \mid a, X_i)S^*(u \mid a, X_i)}\\ & - H(t,a,X_i) - \int_0^\infty \frac{H(u,a,X_i)S^*(t \mid a, X_i)\mathrm{d}M^*_C(u \mid a, X_i)}{S^*_C(u \mid a, X_i)S^{*2}(u \mid a, X_i)}\\ & - \frac{\Delta_i\, Y_i(t)}{S^*_C(t \mid a, X_i)} H_C(t,a,X_i) - \int_0^\infty \frac{H_C(u,a,X_i)S^*(t \mid a, X_i)\mathrm{d}M^*_C(u \mid a, X_i)}{S^{*2}_C(u \mid a, X_i)S^*(u \mid a, X_i)} - \tilde{H}_C(t,a,X_i)\Bigg)\Bigg].\end{aligned}$$

Similarly, we define the following classes of functions:

$$\mathcal{F}^2_\eta = \left\{(d_\eta(x) - d_{\eta^*}(x))\frac{(2a-1)I_{S,i}}{\pi^*_a(x)\pi^{*2}_S(x)} J^*(t,a,x)\left(\frac{\partial \pi^*_S(x)}{\partial \lambda}\right)^T : \|\eta - \eta^*\|_2 < \delta\right\},$$

$$\mathcal{F}^3_\eta = \left\{(d_\eta(x) - d_{\eta^*}(x))\frac{-I_{S,i}}{\pi^{*2}_a(x)\pi^*_S(x)} J^*(t,a,x)\left(\frac{\partial \pi^*_A(x)}{\partial \theta}\right)^T : \|\eta - \eta^*\|_2 < \delta\right\},$$

$$\begin{aligned}\mathcal{F}^4_\eta = \Bigg\{&(d_\eta(x) - d_{\eta^*}(x))\Bigg[I_T\, e(x)(-1)^{a+1}G(t,a,x) + \frac{(2a-1)I_S}{\pi^*_a(x)\pi^*_S(x)}\\ &\times\left(\int_0^\infty \frac{G(t,a,x)\mathrm{d}M^*_C(u \mid a,x)}{S^*_C(u \mid a,x)S^*(u \mid a,x)} - G(t,a,x)\right.\\ &\left. - \int_0^\infty \frac{G(u,a,x)S^*(t \mid a,x)\mathrm{d}M^*_C(u \mid a,x)}{S^*_C(u \mid a,x)S^{*2}(u \mid a,x)}\right)\Bigg] : \|\eta - \eta^*\|_2 < \delta\Bigg\},\end{aligned}$$

$$\begin{aligned}\mathcal{F}^5_\eta = \Bigg\{&(d_\eta(x) - d_{\eta^*}(x))\Bigg[I_T\, e(x)(-1)^{a+1}G(t,a,x) + \frac{(2a-1)I_S}{\pi^*_a(x)\pi^*_S(x)}\\ &\times\left(\int_0^\infty \frac{G(t,a,x)\mathrm{d}M^*_C(u \mid a,x)}{S^*_C(u \mid a,x)S^*(u \mid a,x)} - G(t,a,x)\right.\\ &\left. - \int_0^\infty \frac{G(u,a,x)S^*(t \mid a,x)\mathrm{d}M^*_C(u \mid a,x)}{S^*_C(u \mid a,x)S^{*2}(u \mid a,x)}\right)\Bigg] : \|\eta - \eta^*\|_2 < \delta\Bigg\},\end{aligned}$$

$$\mathcal{F}_\eta^6 = \Bigg\{(d_\eta(x) - d_{\eta^*}(x))\Bigg[\frac{(2a-1)I_S}{\pi_a^*(x)\pi_S^*(x)}\Bigg\{-\frac{\Delta Y(t)}{S_C^*(t\,|\,a,x)}G_C(t,a,x) - \int_0^\infty \frac{G_C(u,a,x)S^*(t\,|\,a,x)\mathrm{d}M_C^*(u\,|\,a,x)}{S_C^{*2}(u\,|\,a,x)S^*(u\,|\,a,x)} + \tilde{G}_C(t,a,x)\Bigg\}\Bigg] : \|\eta-\eta^*\|_2 < \delta\Bigg\},$$

$$\mathcal{F}_\eta^7 = \Bigg\{(d_\eta(x) - d_{\eta^*}(x))\Bigg[\frac{(2a-1)I_S}{\pi_a^*(x)\pi_S^*(x)}\Bigg\{-\frac{\Delta Y(t)}{S_C^*(t\,|\,a,x)}G_C(t,a,x) - \int_0^\infty \frac{G_C(u,a,x)S^*(t\,|\,a,x)\mathrm{d}M_C^*(u\,|\,a,x)}{S_C^{*2}(u\,|\,a,x)S^*(u\,|\,a,x)} + \tilde{G}_C(t,a,x)\Bigg\}\Bigg] : \|\eta-\eta^*\|_2 < \delta\Bigg\},$$

$$\mathcal{F}_\eta^8 = \Bigg\{(d_\eta(x) - d_{\eta^*}(x))\Bigg[\sum_{a=0,1}\Bigg[I_T\, e(x)^{a+1}H(t,a,x) + \frac{(2a-1)I_S}{\pi_a^*(x)\pi_S^*(x)} \times \Bigg(\int_0^\infty \frac{H(t,a,x)\mathrm{d}M_C^*(u\,|\,a,x)}{S_C^*(u\,|\,a,x)S^*(u\,|\,a,x)} - H(t,a,x) - \int_0^\infty \frac{H(u,a,x)S^*(t\,|\,a,x)\mathrm{d}M_C^*(u\,|\,a,x)}{S_C^*(u\,|\,a,x)S^{*2}(u\,|\,a,x)} - \frac{\Delta Y(t)}{S_C^*(t\,|\,a,x)}H_C(t,a,x) - \int_0^\infty \frac{H_C(u,a,x)S^*(t\,|\,a,x)\mathrm{d}M_C^*(u\,|\,a,x)}{S_C^{*2}(u\,|\,a,x)S^*(u\,|\,a,x)} - \tilde{H}_C(t,a,x)\Bigg)\Bigg]\Bigg] : \|\eta-\eta^*\|_2 < \delta\Bigg\}.$$

Let

$$M_2 = \sup\left|\frac{(2a-1)}{\pi_a^*(x)}J^*(t,a,x)\left(\frac{\partial\pi_S^*(x)}{\partial\lambda}\right)^T\right|,$$

where $M_2 \in \mathbb{R}^+$ and the supremum is taken over all the coordinates; and $M_3,\ldots,M_8$ are defined accordingly for $\mathcal{F}_\eta^3,\ldots,\mathcal{F}_\eta^8$. By Assumption 1, 3 and Condition 1, we have that $M_2,\ldots,M_8 < \infty$.

Using the same technique as in **Condition 2.1**, we define the envelop of $\mathcal{F}_\eta^j$ as $F_j = M_j I\{-k_0\delta \le (1,x^T)\eta^* \le k_0\delta\}$ for $j = 2,\ldots,8$, and obtain that

$$\|F_j\|_{P,2} \le \tilde{M}_j\delta^{1/2} < \infty, \quad j = 2,\ldots,8,$$

where $\tilde{M}_2,\ldots,\tilde{M}_8$ are some finite constants, and that $\mathcal{F}_\eta^j$ is a VC class with bounded bracketing entropy $J_{[]}^*(1,\mathcal{F}_\eta^j) < \infty$, for $j = 2,\ldots,8$. By Theorem 11.2 of Kosorok (2008), we obtain

$$\mathbb{E}\left[\sup_{\|\eta-\eta^*\|_2<\delta}\left|\mathbb{G}_N\mathcal{F}_\eta^j\right|\right] \le c_j J_{[]}^*(1,\mathcal{F}_\eta^j)\|F_j\|_{P,2}, \quad j = 2,\ldots,8,$$

where $c_2,\ldots,c_8$ are some finite constants. That is, we have

$$\mathbb{E}\left[\sup_{\|\eta-\eta^*\|_2<\delta}\left|\mathbb{G}_N\mathcal{F}_\eta^8\right|\right] \le \tilde{c}_8\delta^{1/2},$$

and furthermore by Theorem 2.14.5 of van der Vaart and Wellner (1996), we obtain

$$\begin{aligned}\left\{\mathbb{E}\left[\sup_{\|\eta-\eta^*\|_2<\delta}\|\mathbb{G}_n\mathcal{F}_\eta^j\|_2^2\right]\right\}^{1/2} &\leq l_j\left\{\mathbb{E}\left[\sup_{\|\eta-\eta^*\|_2<\delta}|\mathbb{G}_n\mathcal{F}_\eta^j|\right]+\|F_j\|_{P,2}\right\}\\ &\leq l_j\{c_jJ_{[]}^*(1,\mathcal{F}_\eta^j)+1\}\|F_j\|_{P,2}\\ &\leq \tilde{c}_j\delta^{1/2}, \qquad\qquad j=2,\ldots,7,\end{aligned}$$

where $l_2,\ldots,l_7$ and $\tilde{c}_2,\ldots,\tilde{c}_7$ are some finite constants.

By Equation (11), we have that

$$\begin{aligned}(I) &= \mathbb{E}\left[N^{1/2}\sup_{\|\eta-\eta^*\|_2<\delta}\left|\hat{S}(t;\eta)-S_N^*(t;\eta)-\{\hat{S}(t;\eta^*)-S_N^*(t;\eta^*)\}\right|\right]\\ &\leq \mathbb{E}\Bigg[\sup_{\|\eta-\eta^*\|_2<\delta}\Big\{|\mathbb{G}_n\mathcal{F}_\eta^2(\hat{\lambda}-\lambda^*)|+|\mathbb{G}_n\mathcal{F}_\eta^3(\hat{\theta}-\theta^*)|+|\mathbb{G}_n\mathcal{F}_\eta^4(\hat{\beta}_0-\beta_0^*)|+|\mathbb{G}_n\mathcal{F}_\eta^5(\hat{\beta}_1-\beta_1^*)|\\ &\qquad +|\mathbb{G}_n\mathcal{F}_\eta^6(\hat{\alpha}_0-\alpha_0^*)|+|\mathbb{G}_n\mathcal{F}_\eta^7(\hat{\alpha}_1-\alpha_1^*)|+|\mathbb{G}_n\mathcal{F}_\eta^8|\Big\}+o_p(1)\Bigg]\\ &\leq N^{-1/2}\Bigg\{\mathbb{E}\left[\sup_{\|\eta-\eta^*\|_2<\delta}|\mathbb{G}_n\mathcal{F}_\eta^2\cdot N^{1/2}(\hat{\lambda}-\lambda^*)|\right]+\mathbb{E}\left[\sup_{\|\eta-\eta^*\|_2<\delta}|\mathbb{G}_n\mathcal{F}_\eta^3\cdot N^{1/2}(\hat{\theta}-\theta^*)|\right]\\ &\qquad +\mathbb{E}\left[\sup_{\|\eta-\eta^*\|_2<\delta}|\mathbb{G}_n\mathcal{F}_\eta^4\cdot N^{1/2}(\hat{\beta}_0-\beta_0^*)|\right]+\mathbb{E}\left[\sup_{\|\eta-\eta^*\|_2<\delta}|\mathbb{G}_n\mathcal{F}_\eta^5\cdot N^{1/2}(\hat{\beta}_1-\beta_1^*)|\right]\\ &\qquad +\mathbb{E}\left[\sup_{\|\eta-\eta^*\|_2<\delta}|\mathbb{G}_n\mathcal{F}_\eta^6\cdot N^{1/2}(\hat{\alpha}_0-\alpha_0^*)|\right]+\mathbb{E}\left[\sup_{\|\eta-\eta^*\|_2<\delta}|\mathbb{G}_n\mathcal{F}_\eta^7\cdot N^{1/2}(\hat{\alpha}_1-\alpha_1^*)|\right]\Bigg\}\\ &\quad +\mathbb{E}\left[\sup_{\|\eta-\eta^*\|_2<\delta}\left|\mathbb{G}_N\mathcal{F}_\eta^8\right|\right]+o_p(1),\end{aligned}$$

and then by the Cauchy-Schwarz inequality, we obtain

$$
\begin{aligned}
(I) \leq{} & N^{-1/2}\left\{\mathbb{E}[N\|\hat{\lambda}-\lambda^*\|_2^2]\right\}^{1/2}\left\{\mathbb{E}\left[\sup_{\|\eta-\eta^*\|_2<\delta}\|\mathbb{G}_N\mathcal{F}_\eta^2\|_2^2\right]\right\}^{1/2} \\
& + N^{-1/2}\left\{\mathbb{E}[N\|\hat{\theta}-\theta^*\|_2^2]\right\}^{1/2}\left\{\mathbb{E}\left[\sup_{\|\eta-\eta^*\|_2<\delta}\|\mathbb{G}_N\mathcal{F}_\eta^3\|_2^2\right]\right\}^{1/2} \\
& + N^{-1/2}\left\{\mathbb{E}[N\|\hat{\beta}_0-\beta_0^*\|_2^2]\right\}^{1/2}\left\{\mathbb{E}\left[\sup_{\|\eta-\eta^*\|_2<\delta}\|\mathbb{G}_N\mathcal{F}_\eta^4\|_2^2\right]\right\}^{1/2} \\
& + N^{-1/2}\left\{\mathbb{E}[N\|\hat{\beta}_1-\beta_1^*\|_2^2]\right\}^{1/2}\left\{\mathbb{E}\left[\sup_{\|\eta-\eta^*\|_2<\delta}\|\mathbb{G}_N\mathcal{F}_\eta^5\|_2^2\right]\right\}^{1/2} \\
& + N^{-1/2}\left\{\mathbb{E}[N\|\hat{\alpha}_0-\alpha_0^*\|_2^2]\right\}^{1/2}\left\{\mathbb{E}\left[\sup_{\|\eta-\eta^*\|_2<\delta}\|\mathbb{G}_N\mathcal{F}_\eta^6\|_2^2\right]\right\}^{1/2} \\
& + N^{-1/2}\left\{\mathbb{E}[N\|\hat{\alpha}_1-\alpha_1^*\|_2^2]\right\}^{1/2}\left\{\mathbb{E}\left[\sup_{\|\eta-\eta^*\|_2<\delta}\|\mathbb{G}_N\mathcal{F}_\eta^7\|_2^2\right]\right\}^{1/2} \\
& + \mathbb{E}\left[\sup_{\|\eta-\eta^*\|_2<\delta}\left|\mathbb{G}_N\mathcal{F}_\eta^8\right|\right].
\end{aligned}
$$

Let $M_\lambda = \left\{\mathbb{E}[N\|\hat{\lambda}-\lambda^*\|_2^2]\right\}^{1/2}$, and $M_\theta, M_{\beta_0}, M_{\beta_1}, M_{\alpha_0}, M_{\alpha_1}$ are defined accordingly. By Condition 1, we have that $M_\lambda, M_\theta, M_{\beta_0}, M_{\beta_1}, M_{\alpha_0}, M_{\alpha_1} < \infty$, and therefore

$$
(I) \leq N^{-1/2}(M_\lambda\tilde{c}_2 + M_\theta\tilde{c}_3 + M_{\beta_0}\tilde{c}_4 + M_{\beta_1}\tilde{c}_5 + M_{\alpha_0}\tilde{c}_6 + M_{\alpha_1}\tilde{c}_7)\delta^{1/2} + \tilde{c}_8\delta^{1/2}.
$$

In summary, we obtain that, let $N \to \infty$, the centered process satisfies

$$
\begin{aligned}
& \mathbb{E}\left[\sqrt{N}\sup_{\|\eta-\eta^*\|_2<\delta}\left|\hat{S}(t;\eta) - S(t;\eta) - \{\hat{S}(t;\eta^*) - S(t;\eta^*)\}\right|\right] \\
& \leq (I) + (II) \leq (\tilde{c}_1 + \tilde{c}_8)\delta^{1/2}.
\end{aligned} \tag{12}
$$

Let $\phi_N(\delta) = \delta^{1/2}$ and $\alpha = \frac{3}{2} < 2$, thus we have $\frac{\phi_n(\delta)}{\delta^\alpha} = \delta^{-1}$ is decreasing, and α does not depend on N. That is, the second condition holds.

Condition 3 By the facts that $\hat{\eta} \xrightarrow{p} \eta^*$ as $N \to \infty$, and that $\hat{S}(t;\hat{\eta}) \geq \sup_\eta \hat{S}(t;\eta)$, we choose $r_N = N^{1/3}$ such that $r_N^2\phi_N(r_N^{-1}) = N^{2/3}\phi_N(N^{-1/3}) = N^{1/2}$. The third condition holds.

In the end, the three conditions are satisfied with $r_N = N^{1/3}$; thus we conclude that $N^{1/3}\|\hat{\eta} - \eta^*\|_2 = O_p(1)$, which completes the proof of (iii) of Theorem 6.

PART 3. We characterize the asymptotic distribution of $\hat{S}(t;\hat{\eta})$. Since we have

$$
\sqrt{N}\{\hat{S}(t;\hat{\eta}) - S(t;\eta^*)\} = \sqrt{N}\{\hat{S}(t;\hat{\eta}) - \hat{S}(t;\eta^*)\} + \sqrt{N}\{\hat{S}(t;\eta^*) - S(t;\eta^*)\},
$$

we study the two terms in two steps.

Step 3.1 To establish $\sqrt{N}\{\hat{S}(t;\hat{\eta})-\hat{S}(t;\eta^*)\} = o_p(1)$, it suffices to show that $\sqrt{N}\{S(t;\hat{\eta})-S(t;\eta^*)\} = o_p(1)$ and $\sqrt{N}(\hat{S}(t;\hat{\eta}) - \hat{S}(t;\eta^*) - \{S(t;\hat{\eta}) - S(t;\eta^*)\}) = o_p(1)$.

First, as $N^{1/3}\|\hat{\eta} - \eta^*\|_2 = O_p(1)$, we take the second-order Taylor expansion

$$\begin{aligned}
\sqrt{N}\{S(t;\hat{\eta}) - S(t;\eta^*)\} &= \sqrt{N}\left\{S'(\eta^*)\|\hat{\eta} - \eta^*\|_2 + \frac{1}{2}S''(\eta^*)\|\hat{\eta} - \eta^*\|_2^2 + o_p(\|\hat{\eta} - \eta^*\|_2^2)\right\} \\
&= \sqrt{N}\left\{\frac{1}{2}S''(\eta^*)\|\hat{\eta} - \eta^*\|_2^2 + o_p(\|\hat{\eta} - \eta^*\|_2^2)\right\} \\
&= \sqrt{N}\left\{\frac{1}{2}S''(\eta^*)O_p(N^{-2/3}) + o_p(N^{-2/3})\right\} = o_p(1).
\end{aligned}$$

Next, we follow the result (12) obtained in **PART 2**. As $N^{1/3}\|\hat{\eta} - \eta^*\|_2 = O_p(1)$, there exists $\tilde{\delta} = c_9 N^{-1/3}$, where $c_9 < \infty$ is a finite constant, such that $\|\hat{\eta} - \eta^*\|_2 \le \tilde{\delta}$. Therefore we have

$$\begin{aligned}
&\sqrt{N}(\hat{S}(t;\hat{\eta}) - \hat{S}(t;\eta^*) - \{S(t;\hat{\eta}) - S(t;\eta^*)\}) \\
&\le \mathbb{E}\left[\sqrt{N}\sup_{\|\hat{\eta}-\eta^*\|_2<\tilde{\delta}}\left|\hat{S}(t;\hat{\eta}) - S(t;\hat{\eta}) - \{\hat{S}(t;\eta^*) - S(t;\eta^*)\}\right|\right] \\
&\le (\tilde{c}_1 + \tilde{c}_8)\tilde{\delta}^{1/2} = (\tilde{c}_1 + \tilde{c}_8)\sqrt{c_9}N^{-1/6} = o_p(1),
\end{aligned}$$

which yields the result.

Step 3.2 To derive the asymptotic distribution of $\sqrt{n}\{\hat{S}(t;\eta^*) - S(t;\eta^*)\}$, we follow the result (10) obtained in **PART 1** and have that

$$\sqrt{N}\left\{\hat{S}(t;\eta^*) - S(t;\eta^*)\right\} \xrightarrow{D} \mathcal{N}(0, \sigma_{t,1}^2),$$

where $\sigma_{t,1}^2 = \mathbb{E}[(\xi_{1,i}(t;\eta^*) + \xi_{2,i}(t;\eta^*))^2]$. Therefore we obtain in the end

$$\begin{aligned}
\sqrt{N}\{\hat{S}(t;\hat{\eta}) - S(t;\eta^*)\} &= \sqrt{N}\{\hat{S}(t;\hat{\eta}) - \hat{S}(t;\eta^*)\} + \sqrt{N}\{\hat{S}(t;\eta^*) - S(t;\eta^*)\} \\
&= o_p(1) + \sqrt{N}\{\hat{S}(t;\eta^*) - S(t;\eta^*)\} \\
&\xrightarrow{D} \mathcal{N}(0, \sigma_{t,1}^2),
\end{aligned}$$

which completes the proof.

For Corollary 8 where we consider RMST, the proof can follow the same steps as before, and is thus omitted here. ■

Appendix E. Proof of Theorem 7 and Corollary 9

Proof Our proof has three main parts below.

PART 1. Recall that the cross-fitting technique, at a high level as exemplified in Lemma 13, uses sample splitting to avoid bias due to over-fitting. For simplicity, consider that the datasets $\mathcal{O}_s$ and $\mathcal{O}_t$ are randomly split into 2 folds with equal size respectively such that $\mathcal{O}_s = \mathcal{O}_{s,1} \cup \mathcal{O}_{s,2}, \mathcal{O}_t = \mathcal{O}_{t,1} \cup \mathcal{O}_{t,2}$. The extension to K-folds as described in Algorithm

1 is straightforward. Here the subscript CF is omitted to simplify the notation. Define $\mathcal{I}_1 = \mathcal{O}_{s,1} \cup \mathcal{O}_{t,1}, \mathcal{I}_2 = \mathcal{O}_{s,2} \cup \mathcal{O}_{t,2}$, and $N_1 = |\mathcal{I}_1|, N_2 = |\mathcal{I}_2|$. The cross-fitted estimator for the value function under the ITR d_η is

$$\hat{V}(\eta) = \frac{N_1}{N}\hat{V}^{\mathcal{I}_1}(\eta) + \frac{N_2}{N}\hat{V}^{\mathcal{I}_2}(\eta),$$

where

$$\begin{aligned}\hat{V}^{\mathcal{I}_1}(\eta) =& \frac{1}{N_1}\sum_{\mathcal{I}_1}\Bigg\{ I_{T,i}\, e(X_i)\hat{\mu}(d_\eta(X_i), X_i) + \frac{I_{S,i}}{\hat{\pi}_S(X_i)}\frac{I\{A_i = d_\eta(X_i)\}}{\hat{\pi}_d(X_i)} \\ &\times \left(\frac{\Delta_i\, y(U_i)}{\hat{S}_C(U_i \mid A_i, X_i)} - \hat{\mu}(A_i, X_i) + \int_0^\infty \frac{\mathrm{d}\hat{M}_C(u \mid A_i, X_i)}{\hat{S}_C(u \mid A_i, X_i)}\hat{Q}(u, A_i, X_i)\right)\Bigg\},\end{aligned}$$

and the nuisance parameters are estimated from $\mathcal{I}_2$. $\hat{V}^{\mathcal{I}_2}(\eta)$ is defined accordingly.

In this step, we show that

$$\hat{V}(\eta) - V_N(\eta) = o_p(N^{-1/2}),$$

and essentially it suffices to prove that

$$\hat{V}^{\mathcal{I}_1}(\eta) - V_N^{\mathcal{I}_1}(\eta) = o_p(N^{-1/2}),$$

where

$$\begin{aligned}V_N(\eta) =& \frac{1}{N}\sum_{i=1}^{N}\Bigg\{ I_{T,i}\, e(X_i)\mu(d_\eta(X_i), X_i) + \frac{I_{S,i}}{\pi_S(X_i)}\frac{I\{A_i = d_\eta(X_i)\}}{\pi_d(X_i)} \\ &\times \left(\frac{\Delta_i\, y(U_i)}{S_C(U_i \mid A_i, X_i)} - \mu(A_i, X_i) + \int_0^\infty \frac{\mathrm{d}M_C(u \mid A_i, X_i)}{S_C(u \mid A_i, X_i)}Q(u, A_i, X_i)\right)\Bigg\},\end{aligned}$$

and $V_N^{\mathcal{I}_1}(\eta)$ is defined accordingly.

First, we have the following decomposition

$$
\begin{aligned}
&\hat{V}^{\mathcal{I}_1}(\eta) - V_N^{\mathcal{I}_1}(\eta) \\
&= \frac{1}{N_1}\sum_{\mathcal{I}_1}\Bigg\{ I_{T,i}\, e(X_i)(\hat{\mu}(d_\eta(X_i), X_i) - \mu(d_\eta(X_i), X_i)) \\
&\quad + I_{S,i}\left(\frac{1}{\pi_S(X_i)} - \frac{1}{\hat{\pi}_S(X_i)}\right)\frac{I\{A_i = d_\eta(X_i)\}}{\pi_d(X_i)} K(A_i, X_i) \\
&\quad + \frac{I_{S,i} I\{A_i = d_\eta(X_i)\}}{\pi_S(X_i)}\left(\frac{1}{\pi_d(X_i)} - \frac{1}{\hat{\pi}_d(X_i)}\right) K(A_i, X_i) \\
&\quad + \frac{I_{S,i}}{\pi_S(X_i)}\frac{I\{A_i = d_\eta(X_i)\}}{\pi_d(X_i)}(\hat{K}(A_i, X_i) - K(A_i, X_i)) \\
&\quad + I_{S,i} I\{A_i = d_\eta(X_i)\}\left(\frac{1}{\pi_S(X_i)} - \frac{1}{\hat{\pi}_S(X_i)}\right)\left(\frac{1}{\pi_d(X_i)} - \frac{1}{\hat{\pi}_d(X_i)}\right) K(A_i, X_i) \\
&\quad + \frac{I_{S,i} I\{A_i = d_\eta(X_i)\}}{\pi_d(X_i)}\left(\frac{1}{\pi_S(X_i)} - \frac{1}{\hat{\pi}_S(X_i)}\right)(\hat{K}(A_i, X_i) - K(A_i, X_i)) \\
&\quad + \frac{I_{S,i} I\{A_i = d_\eta(X_i)\}}{\pi_S(X_i)}\left(\frac{1}{\pi_d(X_i)} - \frac{1}{\hat{\pi}_d(X_i)}\right)(\hat{K}(A_i, X_i) - K(A_i, X_i)) \\
&\quad + I_{S,i} I\{A_i = d_\eta(X_i)\}\left(\frac{1}{\pi_S(X_i)} - \frac{1}{\hat{\pi}_S(X_i)}\right)\left(\frac{1}{\pi_d(X_i)} - \frac{1}{\hat{\pi}_d(X_i)}\right)(\hat{K}(A_i, X_i) - K(A_i, X_i))\Bigg\},
\end{aligned} \tag{13}
$$

where

$$
\hat{K}(A_i, X_i) = \frac{\Delta_i\, y(U_i)}{\hat{S}_C(U_i \mid A_i, X_i)} - \hat{\mu}(A_i, X_i) + \int_0^\infty \frac{\mathrm{d}\hat{M}_C(u \mid A_i, X_i)}{\hat{S}_C(u \mid A_i, X_i)}\hat{Q}(u, A_i, X_i),
$$

$$
K(A_i, X_i) = \frac{\Delta_i\, y(U_i)}{S_C(U_i \mid A_i, X_i)} - \mu(A_i, X_i) + \int_0^\infty \frac{\mathrm{d}M_C(u \mid A_i, X_i)}{S_C(u \mid A_i, X_i)} Q(u, A_i, X_i).
$$

In summary, the decomposition (13) consists of two types of terms: four mean-zero terms and four product terms. For the mean-zero terms, we utilize the method introduced in Section A.2; since

$$
\mathbb{E}[I_{T,i}\, e(X_i)(\hat{\mu}(d_\eta(X_i), X_i) - \mu(d_\eta(X_i), X_i))] = 0,
$$

by applying Lemma 13, we obtain

$$
\frac{1}{N_1}\sum_{\mathcal{I}_1} I_{T,i}\, e(X_i)(\hat{\mu}(d_\eta(X_i), X_i) - \mu(d_\eta(X_i), X_i)) = o_p(N^{-1/2}).
$$

Similarly we have

$$
\mathbb{E}\left[I_{S,i}\left(\frac{1}{\pi_S(X_i)} - \frac{1}{\hat{\pi}_S(X_i)}\right)\frac{I\{A_i = d_\eta(X_i)\}}{\pi_d(X_i)} K(A_i, X_i)\right] = 0,
$$

so we obtain

$$
\begin{aligned}
&\mathbb{E}\left[\left(\frac{1}{N_1}\sum_{\mathcal{I}_1} I_{S,i}\left(\frac{1}{\pi_S(X_i)}-\frac{1}{\hat{\pi}_S(X_i)}\right)\frac{I\{A_i=d_\eta(X_i)\}}{\pi_d(X_i)}K(A_i,X_i)\right)^2\right] \\
&=\mathbb{E}\left[\mathbb{E}\left[\left(\frac{1}{N_1}\sum_{\mathcal{I}_1} I_{S,i}\left(\frac{1}{\pi_S(X_i)}-\frac{1}{\hat{\pi}_S(X_i)}\right)\frac{I\{A_i=d_\eta(X_i)\}}{\pi_d(X_i)}K(A_i,X_i)\right)^2\middle|\mathcal{I}_2\right]\right] \\
&=\mathbb{E}\left[var\left[\frac{1}{N_1}\sum_{\mathcal{I}_1} I_{S,i}\left(\frac{1}{\pi_S(X_i)}-\frac{1}{\hat{\pi}_S(X_i)}\right)\frac{I\{A_i=d_\eta(X_i)\}}{\pi_d(X_i)}K(A_i,X_i)\middle|\mathcal{I}_2\right]\right] \\
&=\frac{1}{N_1}\mathbb{E}\left[var\left[I_{S,i}\left(\frac{1}{\pi_S(X_i)}-\frac{1}{\hat{\pi}_S(X_i)}\right)\frac{I\{A_i=d_\eta(X_i)\}}{\pi_d(X_i)}K(A_i,X_i)\middle|I_2\right]\right] \\
&\leq \frac{O_p(1)}{N_1}=o_p(\frac{1}{N}).
\end{aligned}
$$

We also have

$$
\mathbb{E}\left[\frac{I_{S,i}I\{A_i=d_\eta(X_i)\}}{\pi_S(X_i)}\left(\frac{1}{\pi_d(X_i)}-\frac{1}{\hat{\pi}_d(X_i)}\right)K(A_i,X_i)\right]=0,
$$

$$
\mathbb{E}\left[\frac{I_{S,i}}{\pi_S(X_i)}\frac{I\{A_i=d_\eta(X_i)\}}{\pi_d(X_i)}(\hat{K}(A_i,X_i)-K(A_i,X_i))\right]=0,
$$

and using the same technique, we conclude that these two mean-zero terms are $o_p(N^{-1/2})$ as well.

The product terms can be handled simply by the Cauchy-Schwarz inequality and the rate of convergence conditions in Assumption 6. Additionally we have the decomposition

as follows

$$
\begin{aligned}
&\frac{1}{N_1}\sum_{\mathcal{I}_1}(\hat{K}(A_i,X_i)-K(A_i,X_i))\\
&=\frac{1}{N_1}\sum_{\mathcal{I}_1}\Bigg\{-(\hat{\mu}(A_i,X_i)-\mu(A_i,X_i))+\frac{1-\Delta_i}{S_C(U_i\,|\,A_i,X_i)}(\hat{Q}(U_i\,|\,A_i,X_i)-Q(U_i\,|\,A_i,X_i))\\
&\quad-\int_0^{U_i}\frac{\lambda_C(u\,|\,A_i,X_i)}{S_C(u\,|\,A_i,X_i)}(\hat{Q}(U_i\,|\,A_i,X_i)-Q(U_i\,|\,A_i,X_i))\mathrm{d}u\\
&\quad+(1-\Delta_i)\left(\frac{1}{\hat{S}_C(U_i\,|\,A_i,X_i)}-\frac{1}{S_C(U_i\,|\,A_i,X_i)}\right)Q(U_i\,|\,A_i,X_i)\\
&\quad+\left(\frac{1}{\hat{S}_C(U_i\,|\,A_i,X_i)}-\frac{1}{S_C(U_i\,|\,A_i,X_i)}\right)\Delta_i\,y(U_i)\\
&\quad-\int_0^{U_i}\left(\frac{\hat{\lambda}_C(u\,|\,A_i,X_i)}{\hat{S}_C(u\,|\,A_i,X_i)}-\frac{\lambda_C(u\,|\,A_i,X_i)}{S_C(u\,|\,A_i,X_i)}\right)Q(U_i\,|\,A_i,X_i)\mathrm{d}u\\
&\quad+(1-\Delta_i)\left(\frac{1}{\hat{S}_C(U_i\,|\,A_i,X_i)}-\frac{1}{S_C(U_i\,|\,A_i,X_i)}\right)(\hat{Q}(U_i\,|\,A_i,X_i)-Q(U_i\,|\,A_i,X_i))\\
&\quad-\int_0^{U_i}\left(\frac{\hat{\lambda}_C(u\,|\,A_i,X_i)}{\hat{S}_C(u\,|\,A_i,X_i)}-\frac{\lambda_C(u\,|\,A_i,X_i)}{S_C(u\,|\,A_i,X_i)}\right)(\hat{Q}(U_i\,|\,A_i,X_i)-Q(U_i\,|\,A_i,X_i))\mathrm{d}u,
\end{aligned}
$$

and similarly we have three mean-zero terms which are $o_p(N^{-1/2})$ by the same technique in Section A.2 and the facts that

$$
\mathbb{E}[\hat{\mu}(A_i,X_i)-\mu(A_i,X_i)]=0,
$$

$$
\begin{aligned}
\mathbb{E}\Bigg[&\frac{1-\Delta_i}{S_C(U_i\,|\,A_i,X_i)}(\hat{Q}(U_i\,|\,A_i,X_i)-Q(U_i\,|\,A_i,X_i))\\
&-\int_0^{U_i}\frac{\lambda_C(u\,|\,A_i,X_i)}{S_C(u\,|\,A_i,X_i)}(\hat{Q}(u\,|\,A_i,X_i)-Q(u\,|\,A_i,X_i))\mathrm{d}u\Bigg]=0,
\end{aligned}
$$

$$
\begin{aligned}
\mathbb{E}\Bigg[&(1-\Delta_i)\left(\frac{1}{\hat{S}_C(U_i\,|\,A_i,X_i)}-\frac{1}{S_C(U_i\,|\,A_i,X_i)}\right)Q(U_i\,|\,A_i,X_i)\\
&+\left(\frac{1}{\hat{S}_C(U_i\,|\,A_i,X_i)}-\frac{1}{S_C(U_i\,|\,A_i,X_i)}\right)\Delta_i\,y(U_i)\\
&-\int_0^{U_i}\left(\frac{\hat{\lambda}_C(u\,|\,A_i,X_i)}{\hat{S}_C(u\,|\,A_i,X_i)}-\frac{\lambda_C(u\,|\,A_i,X_i)}{S_C(u\,|\,A_i,X_i)}\right)Q(U_i\,|\,A_i,X_i)\mathrm{d}u\Bigg]=0,
\end{aligned}
$$

and we can bound the two product terms as well

$$
\begin{aligned}
&\frac{1}{N_1}\sum_{\mathcal{I}_1}\Bigg[(1-\Delta_i)\left(\frac{1}{\hat{S}_C(U_i\,|\,A_i,X_i)}-\frac{1}{S_C(U_i\,|\,A_i,X_i)}\right)(\hat{Q}(U_i\,|\,A_i,X_i)-Q(U_i\,|\,A_i,X_i))\\
&\quad-\int_0^{U_i}\left(\frac{\hat{\lambda}_C(u\,|\,A_i,X_i)}{\hat{S}_C(u\,|\,A_i,X_i)}-\frac{\lambda_C(u\,|\,A_i,X_i)}{S_C(u\,|\,A_i,X_i)}\right)(\hat{Q}(U_i\,|\,A_i,X_i)-Q(U_i\,|\,A_i,X_i))\mathrm{d}u\Bigg]\\
&\leq\left[\frac{1}{N_1}\sum_{\mathcal{I}_1}(1-\Delta_i)\left(\frac{1}{\hat{S}_C(U_i\,|\,A_i,X_i)}-\frac{1}{S_C(U_i\,|\,A_i,X_i)}\right)^2\right]^{1/2}\\
&\quad\times\left[\frac{1}{N_1}\sum_{\mathcal{I}_1}(1-\Delta_i)(\hat{Q}(U_i\,|\,A_i,X_i)-Q(U_i\,|\,A_i,X_i))^2\right]^{1/2}\\
&\quad-\int_0^{U_i}\left[\frac{1}{N_1}\sum_{\mathcal{I}_1}\left(\frac{\hat{\lambda}_C(u\,|\,A_i,X_i)}{\hat{S}_C(u\,|\,A_i,X_i)}-\frac{\lambda_C(u\,|\,A_i,X_i)}{S_C(u\,|\,A_i,X_i)}\right)^2\right]^{1/2}\\
&\quad\times\left[\frac{1}{N_1}\sum_{\mathcal{I}_1}(\hat{Q}(U_i\,|\,A_i,X_i)-Q(U_i\,|\,A_i,X_i))^2\right]^{1/2}\mathrm{d}u\\
&=o_p(N^{-1/2}),
\end{aligned}
$$

which proves that $\frac{1}{N_1}\sum_{\mathcal{I}_1}(\hat{K}(A_i,X_i)-K(A_i,X_i))=o_p(N^{-1/2})$.

Therefore, we conclude that the four product terms in (13) are $o_p(N^{-1/2})$ as well, which completes the proof of (i) in Theorem 7.

PART 2: We show that $N^{1/3}\|\hat{\eta}-\eta^*\|_2=O_p(1)$.

By Assumption 5 (i), $V(\eta)$ is twice continuously differentiable at a neighborhood of η^*; in **PART 1**, we show that $\hat{V}(\eta)=V(\eta)+o_p(1),\forall\eta$; since $\hat{\eta}$ maximizes $\hat{V}(\eta)$, we have that $\hat{V}(\hat{\eta})\geq\sup_\eta\hat{V}(\eta)$, thus by the Argmax theorem, we have $\hat{\eta}\xrightarrow{p}\eta^*$ as $N\to\infty$.

In order to establish the $N^{-1/3}$ rate of convergence of $\hat{\eta}$, we apply Theorem 14.4 (Rate of convergence) of Kosorok (2008), and need to find the suitable rate that satisfies three conditions below.

Condition 1 For every η in a neighborhood of η^* such that $\|\eta-\eta^*\|_2<\delta$, by Assumption 5 (i), we apply the second-order Taylor expansion,

$$
\begin{aligned}
V(\eta)-V(\eta^*)&=V'(\eta^*)\|\eta-\eta^*\|_2+\frac{1}{2}V''(\eta^*)\|\eta-\eta^*\|_2^2+o(\|\eta-\eta^*\|_2^2)\\
&=\frac{1}{2}V''(\eta^*)\|\eta-\eta^*\|_2^2+o(\|\eta-\eta^*\|_2^2),
\end{aligned}
$$

and as $V''(\eta^*)<0$, there exists $c_{10}=-\frac{1}{2}V''(\eta^*)>0$ such that $V(\eta)-V(\eta^*)\leq-c_{10}\|\eta-\eta^*\|_2^2$.

Condition 2 For all N large enough and sufficiently small δ, we consider the centered process $\hat{V} - V$, and have that

$$\begin{aligned}
&\mathbb{E}\left[\sqrt{N}\sup_{\|\eta-\eta^*\|_2<\delta}\left|\hat{V}(\eta)-V(\eta)-\{\hat{V}(\eta^*)-V(\eta^*)\}\right|\right]\\
&=\mathbb{E}\left[\sqrt{N}\sup_{\|\eta-\eta^*\|_2<\delta}\left|\hat{V}(\eta)-V_n(\eta)+V_n(\eta)-V(\eta)-\{\hat{V}(\eta^*)-V_n(\eta^*)+V_n(\eta^*)-V(\eta^*)\}\right|\right]\\
&\leq\mathbb{E}\left[\sqrt{N}\sup_{\|\eta-\eta^*\|_2<\delta}\left|\hat{V}(\eta)-V_n(\eta)-\{\hat{V}(\eta^*)-V_n(\eta^*)\}\right|\right] \qquad (I)\\
&\quad+\mathbb{E}\left[\sqrt{N}\sup_{\|\eta-\eta^*\|_2<\delta}\left|V_n(\eta)-V(\eta)-\{V_n(\eta^*)-V(\eta^*)\}\right|\right] \qquad (II)
\end{aligned}$$

It follows from the result in **PART 1** that $(I) = o_p(1)$. To bound (II), we have

$$\begin{aligned}
&V_n(\eta)-V_n(\eta^*)\\
&=\frac{1}{N}\sum_{i=1}^{N}(d_\eta(X_i)-d_{\eta^*}(X_i))\times\left(I_{T,i}\,e(X_i)(\mu(1,X_i)-\mu(0,X_i))+\frac{(2A_i-1)I_{S,i}}{\pi_{A_i}(X_i)\pi_S(X_i)}K(A_i,X_i)\right).
\end{aligned}$$

Define a class of functions

$$\mathcal{F}_\eta^9=\left\{(d_\eta(x)-d_{\eta^*}(x))\times\left(I_T\,e(x)(\mu(1,x)-\mu(0,x))+\frac{(2a-1)I_S}{\pi_a(x)\pi_S(x)}K(a,x)\right):\|\eta-\eta^*\|_2<\delta\right\},$$

and let $M_9 = \sup\left|I_T\,e(x)(\mu(1,x)-\mu(0,x))+\frac{(2a-1)I_S}{\pi_a(x)\pi_S(x)}K(a,x)\right|$. By Assumption 1, 3 and Condition 1, we have that $M_9 < \infty$. Using the same technique as in Section D.2 **Condition 2.1**, we define the envelop of $\mathcal{F}_\eta^9$ as $F_9 = M_9 I\{-k_0\delta \leq (1, x^T)\eta^* \leq k_0\delta\}$, and obtain that $\|F_9\|_{P,2} \leq \tilde{M}_9\delta^{1/2} < \infty$, where $\tilde{M}_9$ is a finite constant, and that $\mathcal{F}_\eta^9$ is a VC class with bounded entropy $J_{[]}^*(1, \mathcal{F}_\eta^9) < \infty$. By Theorem 11.2 of Kosorok (2008), we obtain

$$\mathbb{E}\left[\sup_{\|\eta-\eta^*\|_2<\delta}\left|\mathbb{G}_N\mathcal{F}_\eta^9\right|\right]\leq\tilde{c}_9\delta^{1/2},$$

where $\tilde{c}_9$ is a finite constant. Therefore, we obtain

$$\begin{aligned}
(II)&=\mathbb{E}\left[\sqrt{N}\sup_{\|\eta-\eta^*\|_2<\delta}\left|V_N(\eta)-V(\eta)-\{V_N(\eta^*)-V(\eta^*)\}\right|\right]\\
&=\mathbb{E}\left[\sup_{\|\eta-\eta^*\|_2<\delta}\left|\mathbb{G}_n\mathcal{F}_\eta^9\right|\right]\leq\tilde{c}_9\delta^{1/2}.
\end{aligned}$$

In summary, we obtain that the centered process satisfies

$$\begin{aligned}
&\mathbb{E}\left[\sqrt{N}\sup_{\|\eta-\eta^*\|_2<\delta}\left|\hat{S}(t;\eta)-S(t;\eta)-\{\hat{S}(t;\eta^*)-S(t;\eta^*)\}\right|\right]\\
&\leq (I)+(II)\leq\tilde{c}_9\delta^{1/2}.
\end{aligned} \qquad (14)$$

Let $\phi_N(\delta) = \delta^{1/2}$ and $\alpha = \frac{3}{2} < 2$, thus we have $\frac{\phi_n(\delta)}{\delta^\alpha} = \delta^{-1}$ is decreasing, and α does not depend on N. That is, the second condition holds.

Condition 3 By the facts that $\hat{\eta} \xrightarrow{p} \eta^*$ as $N \to \infty$, and that $\hat{S}(t;\hat{\eta}) \geq \sup_\eta \hat{S}(t;\eta)$, we choose $r_N = N^{1/3}$ such that $r_N^2 \phi_N(r_N^{-1}) = N^{2/3}\phi_N(N^{-1/3}) = N^{1/2}$. The third condition holds.

In the end, the three conditions are satisfied with $r_N = N^{1/3}$; thus we conclude that $N^{1/3}\|\hat{\eta} - \eta^*\|_2 = O_p(1)$, which completes the proof of (ii) in Theorem 7.

PART 3: We characterize the asymptotic distribution of $\hat{V}(\hat{\eta})$. Since we have

$$\sqrt{N}\{\hat{V}(\hat{\eta}) - V(\eta^*)\} = \sqrt{N}\{\hat{V}(\hat{\eta}) - \hat{V}(\eta^*)\} + \sqrt{N}\{\hat{V}(\eta^*) - V(t;\eta^*)\},$$

we study the two terms in two steps.

Step 3.1 To establish $\sqrt{N}\{\hat{V}(\hat{\eta}) - \hat{V}(\eta^*)\} = o_p(1)$, it suffices to show that $\sqrt{N}\{V(\hat{\eta}) - V(\eta^*)\} = o_p(1)$ and $\sqrt{N}(\hat{V}(\hat{\eta}) - \hat{V}(\eta^*) - \{V(\hat{\eta}) - V(\eta^*)\}) = o_p(1)$.

First, as $N^{1/3}\|\hat{\eta} - \eta^*\|_2 = O_p(1)$, we take the second-order Taylor expansion

$$\begin{aligned}
\sqrt{N}\{V(\hat{\eta}) - V(\eta^*)\} &= \sqrt{N}\left\{V'(\eta^*)\|\hat{\eta} - \eta^*\|_2 + \frac{1}{2}V''(\eta^*)\|\hat{\eta} - \eta^*\|_2^2 + o_p(\|\hat{\eta} - \eta^*\|_2^2)\right\} \\
&= \sqrt{N}\left\{\frac{1}{2}V''(\eta^*)\|\hat{\eta} - \eta^*\|_2^2 + o_p(\|\hat{\eta} - \eta^*\|_2^2)\right\} \\
&= \sqrt{N}\left\{\frac{1}{2}V''(\eta^*)O_p(N^{-2/3}) + o_p(N^{-2/3})\right\} = o_p(1).
\end{aligned}$$

Next, we follow the result (14) obtained in **PART 2**. As $N^{1/3}\|\hat{\eta} - \eta^*\|_2 = O_p(1)$, there exists $\tilde{\delta}_2 = c_{11}N^{-1/3}$, where $c_{11} < \infty$ is a finite constant, such that $\|\hat{\eta} - \eta^*\|_2 \leq \tilde{\delta}_2$. Therefore we have

$$\begin{aligned}
&\sqrt{N}(\hat{V}(\hat{\eta}) - \hat{V}(\eta^*) - \{V(\hat{\eta}) - V(\eta^*)\}) \\
&\leq \mathbb{E}\left[\sqrt{N} \sup_{\|\hat{\eta} - \eta^*\|_2 < \tilde{\delta}_2} \left|\hat{V}(\hat{\eta}) - V(\hat{\eta}) - \{\hat{V}(\eta^*) - V(\eta^*)\}\right|\right] \\
&\leq \tilde{c}_9\tilde{\delta}^{1/2} = \tilde{c}_9\sqrt{c_{11}}N^{-1/6} = o_p(1),
\end{aligned}$$

which yields the result.

Step 3.2 To derive the asymptotic distribution of $\sqrt{N}\{\hat{V}(\eta^*) - V(\eta^*)\}$, we follow the result obtained in **PART 1** that $\hat{V}(\eta^*) = V_N(\eta^*) + o_p(N^{-1/2})$, and thus

$$\sqrt{N}\left\{\hat{V}(\eta^*) - V(\eta^*)\right\} \xrightarrow{D} \mathcal{N}(0, \sigma_2^2),$$

where $\sigma_2^2 = \mathbb{E}[\phi_{d_{\eta^*}}^2]$ is the semiparametric efficiency bound.

Therefore we obtain in the end

$$\begin{aligned}
\sqrt{N}\{\hat{V}(\hat{\eta}) - v(\eta^*)\} &= \sqrt{N}\{\hat{V}(\hat{\eta}) - \hat{V}(\eta^*)\} + \sqrt{N}\{\hat{V}(\eta^*) - V(\eta^*)\} \\
&= o_p(1) + \sqrt{N}\{\hat{V}(\eta^*) - V(\eta^*)\} \\
&\xrightarrow{D} \mathcal{N}(0, \sigma_2^2),
\end{aligned}$$

which completes the proof of Theorem 7 and Corollary 9. ■

Appendix F. Analysis of divergent sample sizes

In this section, we analyze the asymptotic properties of the ACW estimator

$$\begin{aligned}\hat{V}(d) =& \frac{1}{m}\sum_{i=n+1}^{n+m}\hat{\mu}(A=d(X_i),X_i)\\ &+\frac{1}{n}\sum_{i=1}^{n}\frac{I\{A_i=d(X_i)\}}{\hat{\pi}_S(X_i)\hat{\pi}_d(X_i)}\left\{\frac{\Delta_i\, y(U_i)}{\hat{S}_C(U_i\mid A_i,X_i)}-\hat{\mu}(A_i,X_i)+\int_0^\infty\frac{\mathrm{d}\hat{M}_C(u\mid A_i,X_i)}{\hat{S}_C(u\mid A_i,X_i)}\hat{Q}(u,A_i,X_i)\right\},\end{aligned}$$

when the sample sizes n, m diverge.

Note that we solve the following estimation equations to obtain the calibration weights:

$$\frac{1}{m}\sum_{i=n+1}^{n+m}\{g(X_i)-\mu_g\}=0,$$

$$\frac{1}{n}\sum_{i=1}^{n}\exp\{\lambda^T g(X_i)\}\{g(X_i)-\mu_g\}=0.$$

When m diverges faster than n, we characterize $\sqrt{n}\{\hat{V}(d)-V(d)\}$. First we have that $\mu_g=E[g(X)]$, and $\hat{\lambda}$ is the solution to the estimation equation $n^{-1}\sum_{i=1}^{n}\exp\{\lambda^T g(X_i)\}\{g(X_i)-\mu_g\}=0$. Thus we obtain that

$$\begin{aligned}\sqrt{n}\{\hat{V}(d)-V(d)\} =& \frac{1}{m}\sum_{i=n+1}^{n+m}\sqrt{n}\{\hat{\mu}(A=d(X_i),X_i)-V(d)\}\\ &+\frac{1}{\sqrt{n}}\sum_{i=1}^{n}\phi(O_i)q_i,\end{aligned}$$

where $\phi(O_i)$ is the influence function. Finally, we conclude that $\sqrt{n}\{\hat{V}(d)-V(d)\}\rightarrow\mathcal{N}(0,\sigma_1^2)$, where $\sigma_1^2=E[\phi^2(O)q]$.

When n diverges faster than m, we characterize $\sqrt{m}\{\hat{V}(d)-V(d)\}$. First we have that $\hat{\lambda}$ is the solution to the estimation equation

$$\frac{1}{m}\sum_{i=n+1}^{n+m}\{g(X_i)M_{S,1}(\lambda)-M_{S,2}(\lambda)\}=0,$$

where $M_{S,1}(\lambda)=E[I_S\exp\{\lambda^T g(X)\}]$, and $M_{S,2}(\lambda)=E[I_S\exp\{\lambda^T g(X)\}g(X)]$.

Thus we obtain that

$$\begin{aligned}\sqrt{m}\{\hat{V}(d)-V(d)\} =& \frac{1}{\sqrt{m}}\sum_{i=n+1}^{n+m}\{\hat{\mu}(A=d(X_i),X_i)-V(d)\}\\ &+\frac{1}{n}\sum_{i=1}^{n}\sqrt{m}\phi(O_i)q_i,\end{aligned}$$

Finally, we conclude that $\sqrt{m}\{\hat{V}(d)-V(d)\}\rightarrow\mathcal{N}(0,\sigma_2^2)$, where $\sigma_2^2=E[(\mu(d(X),X)-V(d))^2]$.

Appendix G. Proof of Theorem 10 and Theorem 11

Proof When the source and target populations have the same distributions, both $\hat{V}_{DR}(\eta)$ and $\hat{V}_{CF}(\eta)$ converge to $V(\eta)$. The asymptotic variance of $\hat{V}_{DR}(\eta)$ is

$$\begin{aligned}\sigma_{DR}^2 &= \mathbb{E}\left[\frac{I_S}{\mathbb{P}(I_S=1)}\left(\mu(d(X),X)+\frac{I\{A=d(X)\}}{\pi_d(X)}K(A,X)-V(\eta)\right)^2\right]\\ &= \mathbb{E}\left[\frac{I_S}{\mathbb{P}(I_S=1)}\left(\mu^2(d(X),X)+\frac{I\{A=d(X)\}}{\pi_d^2(X)}K^2(A,X)-V^2(\eta)\right.\right.\\ &\qquad +\frac{2I\{A=d(X)\}}{\pi_d(X)}K(A,X)\mu(d(X),X)-2\mu(d(X),X)V(\eta)\\ &\qquad \left.\left.-\frac{2I\{A=d(X)\}}{\pi_d(X)}K(A,X)V(\eta)\right)\right],\end{aligned}$$

while the asymptotic variance of $\hat{V}_{CF}(\eta)$ is

$$\begin{aligned}\sigma_{CF}^2 &= \mathbb{E}\left[\left(I_T\, e(X)\mu(d(X),X)+\frac{I_S\, I\{A=d(X)\}}{\pi_S(X)\pi_d(X)}K(A,X)-V(\eta)\right)^2\right]\\ &= \mathbb{E}\left[\left(I_T\, e^2(X)\mu^2(d(X),X)+\frac{I_S\, I\{A=d(X)\}}{\pi_S^2(X)\pi_d^2(X)}K^2(A,X)-V^2(\eta)\right.\right.\\ &\qquad \left.\left.-2I_T\, e^2(X)\mu(d(X),X)V(\eta)-\frac{2I_S\, I\{A=d(X)\}}{\pi_S(X)\pi_d(X)}K(A,X)V(\eta)\right)\right],\end{aligned}$$

where

$$K(A,X)=\frac{\Delta\, y(U)}{S_C(U\,|\,A,X)}-\mu(A,X)+\int_0^\infty \frac{\mathrm{d}M_C(u\,|\,A,X)}{S_C(u\,|\,A,X)}Q(u,A,X).$$

Since we have that

$$\mathbb{E}\left[\frac{I_S}{\mathbb{P}(I_S=1)}\frac{2I\{A=d(X)\}}{\pi_d(X)}K(A,X)\mu(d(X),X)\right]=0,$$

and for

$$B\in\left\{\mu^2(d(X),X),\frac{I\{A=d(X)\}}{\pi_d^2(X)}K^2(A,X),\mu(d(X),X)V(\eta),\frac{I\{A=d(X)\}}{\pi_d^2(X)}K(A,X)V(\eta)\right\},$$

we also have that

$$\mathbb{E}\left[\frac{I_S}{\mathbb{P}(I_S=1)}B\right]=\mathbb{E}[I_T\, e(X)B]=\mathbb{E}\left[\frac{I_S}{\pi_S(X)}B\right],$$

we conclude that $\sigma_{DR}^2=\sigma_{CF}^2$.

By the law of iterated expectations, the value function $V_d=\mathbb{E}[y(T(d))]=\mathbb{E}_X[\mathbb{E}[y(T(d))\,|\,X]]$. When there is no restriction on the class of ITRs, the true optimal ITR is

$$\begin{aligned}d^{**}(X) &= \arg\max_d V_d=\arg\max_d \mathbb{E}_X[\mathbb{E}[y(T(d))\,|\,X]]\\ &= I\{\mathbb{E}[y(T(1))\,|\,X]>\mathbb{E}[y(T(0))\,|\,X]\}.\end{aligned}$$

That is, the optimal ITR does not depend on the covariate distributions, but only the bilp function which is the same in both the source and target populations by Assumption 2. Thus both the maximizers of $\hat{V}_{DR}(\eta)$ and $\hat{V}_{CF}(\eta)$ converge to the true population parameter η^{**}. However, $\hat{V}_{DR}(\eta)$ is biased since the expectation $\mathbb{E}_X$ is taken with respect to the source population. ■

Appendix H. Additional simulations

We first investigate the performance of the cross-fitted ACW estimator with different sample sizes $(N, m) = (5 \times 10^4, 2000), (1 \times 10^5, 4000), (2 \times 10^5, 8000), (4 \times 10^5, 16000), (6 \times 10^5, 24000), (8 \times 10^5, 32000)$. Figure 3 and Table 3 report the results from 200 Monte Carlo replications. The variance is computed using the EIF.

Figure 3: Boxplot of estimated value by ACW estimator with different sample sizes.

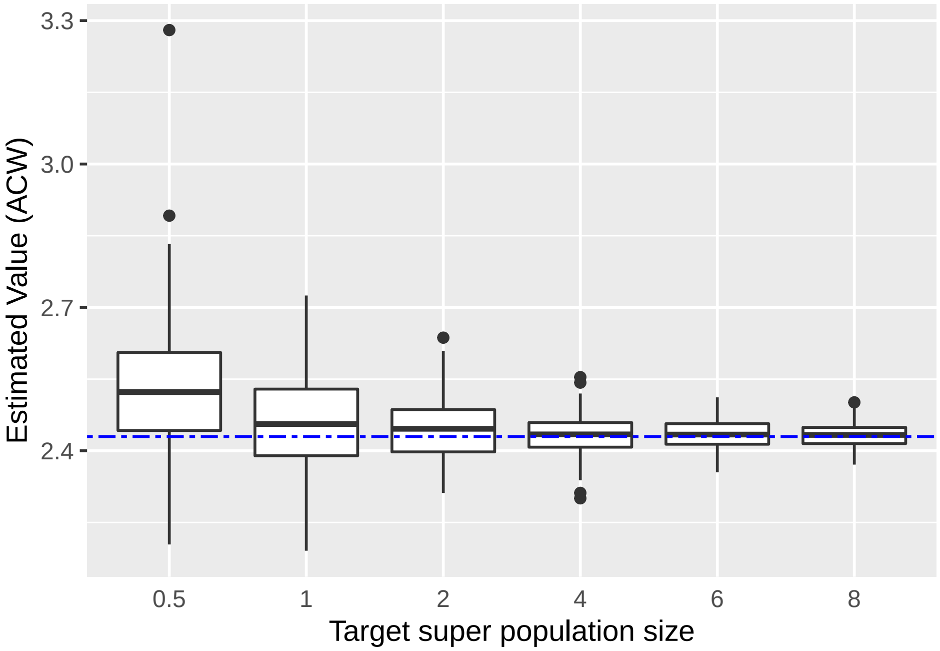

Appendix I. Details of real data analysis

There are around 0.5% and 1.6% missing values in the RCT and OS data, respectively. We use the `mice` function in the `R` package `mice` (van Buuren and Groothuis-Oudshoorn, 2011) to impute the missing values.

Motivated by the clinical practice and existing work in the medical literature, we consider ITRs that depend on the following five variables:

Table 3: Numeric results of the ACW estimator. Bias is the empirical bias of point estimates; SD is the empirical standard deviation of point estimates; SE is the average of standard error estimates; CP is the empirical coverage probability of the 95% Wald confidence intervals.

$n; m(\times 10^3)$	$\sim 780; 2$	$\sim 1560; 4$	$\sim 3120; 8$	$\sim 6240; 16$	$\sim 9360; 24$	$\sim 12480; 32$
Bias	0.1041	0.0253	0.0134	0.0046	0.0031	0.0030
SD	0.1394	0.0985	0.0635	0.0419	0.0317	0.0267
SE	0.1611	0.0942	0.0627	0.0417	0.0330	0.0284
CP(%)	97.5	93.5	96.0	94.5	97.5	97.0

- AGE, SEX and Sequential Organ Failure Assessment (SOFA) score: these three baseline variables are well related to mortality in ICUs, so we consider them as important risk factors.
- Acute Kidney Injury Network (AKIN) score: Jaber et al. (2018) observed that the infusion of sodium bicarbonate improved survival outcomes and mortality rate in critically ill patients with severe metabolic acidemia and acute kidney injury. In the observational data, the AKIN score was not recorded, so we computed the score using serum creatinine measurement (Závada et al., 2010).
- SEPSIS: we consider the presence of sepsis as a risk factor because it is the main condition associated with severe acidemia at the arrival in ICU. The effect of sodium bicarbonate infusion on patients with acidema and acute kidney injury was also observed in septic patients (Zhang et al., 2018b).
